# Comprehensive spatial distribution of tropical fish assemblages from multifrequency acoustics and video fulfils the island mass effect framework

**DOI:** 10.1038/s41598-022-12409-9

**Published:** 2022-05-24

**Authors:** Julie Salvetat, Nicolas Bez, Jeremie Habasque, Anne Lebourges-Dhaussy, Cristiano Lopes, Gildas Roudaut, Monique Simier, Paulo Travassos, Gary Vargas, Arnaud Bertrand

**Affiliations:** 1 Pós-Graduação em Recursos Pesqueiros e Aquicultura, Universidade Federal Rural de Pernambuco, Rua Dom Manoel de Medeiros, s/n, Dois Irmãos, Recife, PE 52171-900 Brazil; 2 MARBEC, Univ Montpellier, CNRS, IRD, Ifremer, Sète, France; 3 Institut de Recherche pour le Développement, Sète, France; 4 IRD, LEMAR UBO/CNRS/IRD/Ifremer, Plouzané, France; 5 Laboratório de Oceanografia Física Estuarina e Costeira, Depto. Oceanografia, Universidade Federal de Pernambuco, Av. Prof. Moraes Rego, 1235-Cidade Universitária, Recife, PE 50670-901 Brazil

**Keywords:** Ocean sciences, Ecology

## Abstract

Tropical marine ecosystems are highly biodiverse and provide resources for small-scale fisheries and tourism. However, precise information on fish spatial distribution is lacking, which limits our ability to reconcile exploitation and conservation. We combined acoustics to video observations to provide a comprehensive description of fish distribution in a typical tropical environment, the Fernando de Noronha Archipelago (FNA) off Northeast Brazil. We identified and classified all acoustic echoes into ten fish assemblage and two triggerfish species. This opened up the possibility to relate the different spatial patterns to a series of environmental factors and the level of protection. We provide the first biomass estimation of the black triggerfish *Melichthys niger*, a key tropical player. By comparing the effects of euphotic and mesophotic reefs we show that more than the depth, the most important feature is the topography with the shelf-break as the most important hotspot. We also complete the portrait of the island mass effect revealing a clear spatial dissymmetry regarding fish distribution. Indeed, while primary productivity is higher downstream, fish concentrate upstream. The comprehensive fish distribution provided by our approach is directly usable to implement scientific-grounded Marine Spatial Planning.

## Introduction

Tropical marine ecosystems hold major biodiversity hotspots^1^ and provide a significant share of global fish catch^2^. Meanwhile, they are increasingly threatened by anthropic pressure including overfishing, global change, invasive species introduction, habitats destruction and pollution^3^. In particular, on-going global ocean warming is expected to severely affect species distribution, abundance and extinction rates but also trophic interactions and entire food webs balance^4,5^. These threats are critical especially for human populations that rely heavily on marine resources and depend on small-scale fisheries (SSF) or tourism for their livelihoods such as tropical developing states or small tropical islands^6–8^.

Tropical coastal environments form a mosaic of interconnected mega-habitats extending from the shoreline to the open ocean. This complex structure greatly influences the dynamics of fish assemblages^9^. In recent years, mesophotic reef ecosystems (MREs) have gained attention^10–14^, not least because their depth may offer protection from anthropic stressors^14,15^. MREs occur in tropical and subtropical regions and are characterized by the presence of light-dependent corals and associated fauna at depths below 30–40 m extending to 150 m in areas with high water clarity^12,13,16^. MREs are known hot spots of tropical fish diversity and host fish communities ecologically distinct from shallow water reefs^17^. The mesophotic zone usually encompasses the shelf-break, a transition area from shelf to ocean characterised by a rapid change in the topography with a steep slope. The stiffness of the slope is associated with turbulent mixing enhancing primary productivity and therefore attracting prey and predators^18–20^. It concentrates diverse fishing resources over a relatively narrow area, sustaining important multispecific reef fisheries^21–24^. However, so far few study actually quantified the relative importance of mesophotic reefs for fish and/or in comparison to euphotic reefs, in particular because consistent observations extending from the shoreline to the shelf-break are lacking^25^.

Oceanic islands and shallow seamounts also act as topographic anomalies that trigger complex physical processes increasing primary production and concentrating higher trophic levels. This phenomenon, known as the Island Mass Effect (IME^26^) is originated by the turbulence created by the island bathymetry, which uplift nutrient-rich water into the photic zone, enhancing primary production^27^. Oceanic islands and shallow seamounts are important environments for maintaining local biodiversity and non-resident migrating top predatory species^28^. IME aggregative effect on top predators supports commercial, artisanal and recreational fisheries^29,30^, which play an important role in the local socio-economic life of insular populations^31^. So far, most studies on the IME focused on physical-biogeochemical processes^32,33^. They showed that primary productivity is most enhanced on the leeward side of islands^30,34^. However, since fewer studies focused on higher trophic levels, the response of fish is generally depicted as symmetrical around islands^27^. No studies, for instance, determined if fish follow the pattern of primary productivity and concentrate downstream of islands.

Yet, this kind of knowledge is essential to assist decision making in conservation policies to protect biodiversity and the sustainability of fishing and diverse marine uses. Protective management is generally achieved through the creation of Marine Protected Areas (MPAs) delineating permitted and non-permitted zones according to pre-defined management objectives^35^. However, in some cases, the consequences of establishing MPAs are not adequately thought out, and a poorly planned MPA can be detrimental for local populations that rely on marine resources^36^. Indeed the decision support tools used to design MPAs rely on available data. To coherently manage the use of maritime space and achieve ecological, economic and social objectives, Marine Spatial Planning (MSP) is increasingly used as a strategic alternative aiming at integrating MPAs in a broader context^37^. MSP is a complex process requiring the use of optimization solvers that ultimately requires large quantities of spatially explicit cross-disciplinary knowledge and data (ecological, legal, social, economic)^38^. One of the main challenges to improve knowledge of tropical ecosystems and their resources and implement MSP is thus the data collection^39^.

Comprehensive monitoring is required to provide ground information for sustainable management^40,41^. Fish assemblage data are often used to help understanding how human activities influence marine ecosystems^42,43^ or as a measure of ecosystem health^44^ and as a basis for managerial decisions^45^. A variety of methods is used to assess tropical fish populations, including fishing gears or visual observations, each presenting its own pros and cons^46^. Fishery-dependent methods provide long time-series, wide spatiotemporal coverage but are biased by, among other, gear selectivity^47^. Scientific fish catches are more reliant but have a limited spatiotemporal coverage^47^*.* Tropical reef fish communities are also classically described via direct in situ observations through diver-based underwater visual census (UVC)^48^. Scuba diving is constrained by a set of limitations including underwater time and maximal diving depth and visibility^49–51^. As a result, most UVC-based studies are restricted to near shore shallow waters and provide punctual small-scale information whereas species richness and patterns of distribution is heavily influenced by the range of the sampling area^52^. To overcome part of these limitations and bias, underwater video techniques are increasingly used, whether stationary or towed, remotely operated or autonomous, baited or not^53^. The use of video increases sampling range and is more time efficient than diver-based observation^54^ but each technique has different limitations and combining underwater video and other sampling methods is therefore recommended^55^.

Active acoustics, in particular multi-frequency, is a powerful tool allowing simultaneous and continuous observation of the distribution of a variety of marine communities and abiotic characteristics^56–58^. Acoustics range of observation reaches several hundreds of meters below the surface, which allows prospecting the pelagic domain^59^. However, the ability to discriminate acoustically among taxa remains coarse and works best in relatively low-diversity temperate systems with a few well-defined and acoustically distinct groups^56^. Acoustic species discrimination remains challenging in highly diverse tropical ecosystems. Moreover, acoustics methods needs to be coupled with other observational methods to perform species identification, classically extractive one such as trawls and nets^60^. But trawling can be destructive and is not always possible to operate in topographically complex environments or in MPAs^61–63^. To lift out this lock, the combination of acoustic methods with non-extractive optical methods has emerged. These methods were mostly applied in temperate water^64–69^ whereas to date, only few studies focused on tropical waters^70–72^.

**Figure 1:**
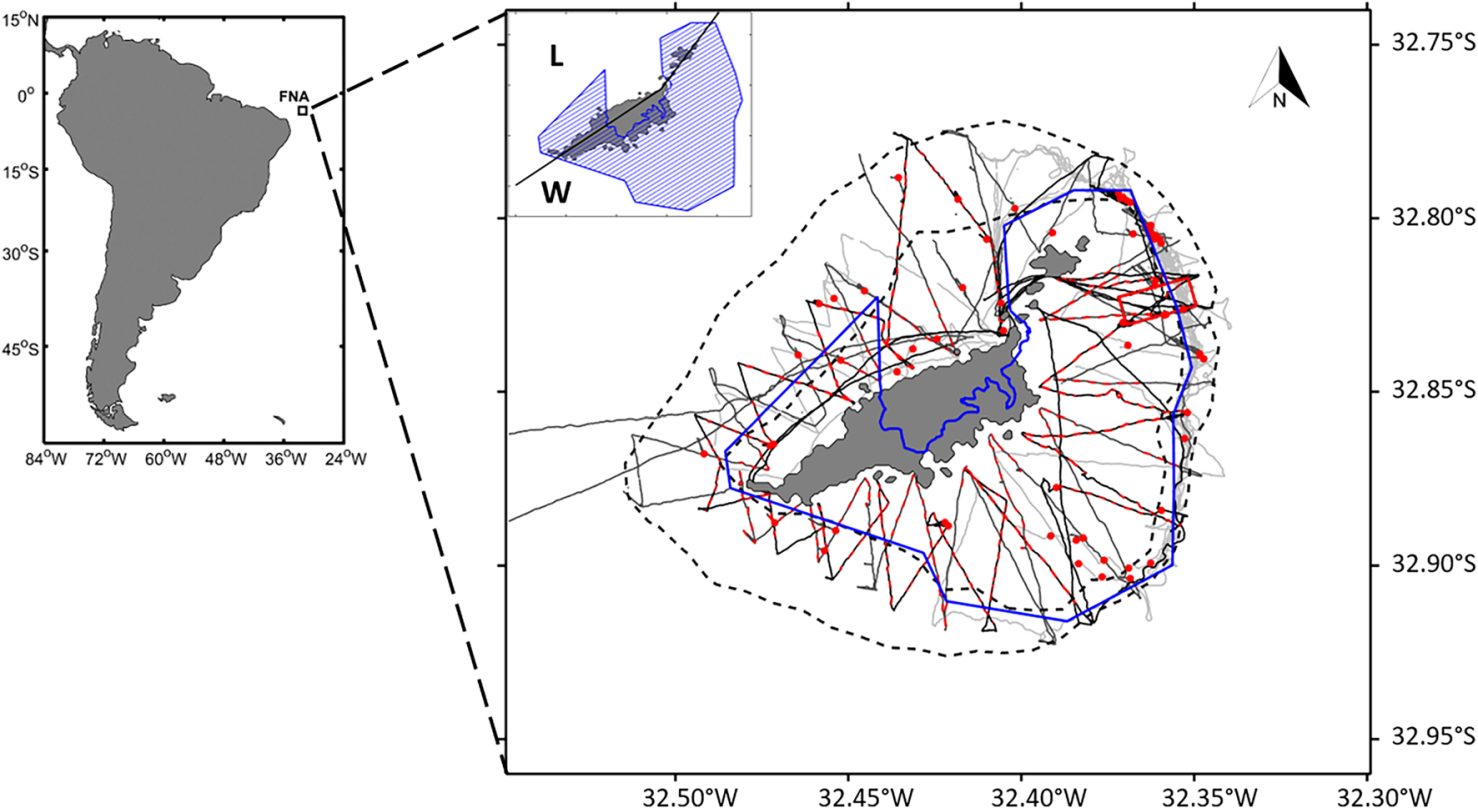
Fernando de Noronha archipelago (FNA) (03°51′S, 32°25′W). The blue line delimits the no take MPA PARNAMAR. Acoustic transects are depicted by light grey (FAROFA 1), dark grey (FAROFA 2) and black lines (FAROFA 3); video transects by red dashed lines and video stations by red dots. The black dashed lines depict the 50 and 300 m isobaths. The insert on the main map show the PARNAMAR hatched in blue and the black solid line separates the leeward side (L) and the windward side (W). Map was created by the authors using Matlab R2018b (https://fr.mathworks.com/) and m_map mapping package^84^.

In this context, we combine multifrequency acoustic and video observation to provide a comprehensive vision of fish distribution around a tropical oceanic marine ecosystem. The study area, Fernando de Noronha Archipelago (FNA) located ~ 350 km off the coast of Brazil (Fig. 1), is a typical low productivity and high biodiversity system^34,73,74^, representative of tropical ecosystems. Like many other tropical small islands, the local population of FNA relies on artisanal fisheries for protein income^75^ and the economic activity is mainly based on tourism. Tourism generates demographic pressure and all its externalities, amplifying the demand for fish but also enhancing marine related activities such as recreational fishing or diving^76,77^. Beside, FNA is protected by a series of legal instruments regulating the uses of the marine environment and marine resources. Indeed, FNA is bathing in an Environmental Protection Area (EPA) where sustainable use of marine resources and tourism is allowed. The EPA includes a smaller no-take MPA, the “National Marine Park of Fernando de Noronha (PARNAMAR)”, covering about 70% of the main island and the coastline from the shore to the 50 m isobaths^78^.

On the base of three surveys combining multi-frequency active acoustics and underwater videos, we propose to address the questions identified above. Specifically our work includes a series of objectives. First, we aim at providing a comprehensive description of the distribution of the acoustic fish biomass and fish assemblages in FNA. Second, we propose to perform the biomass estimation of the most observed fish, the black triggerfish *Melichthys niger*. Third, we propose to quantify the relative importance of mesophotic reefs for fish in comparison to euphotic reefs. Four, on this background, we propose to complete the portrait of the IME. Finally, we propose to discuss how the comprehensive information gained by such approach can be usable to implement scientific-grounded MSP.

## Material and methods

Data were collected during three ‘Fish Acoustics around Fernando de Noronha’ (FAROFA) surveys performed on-board a 10-m-long sport fishing boat (see Supplementary Fig. S1 online) in September 15–21, 2017 (FAROFA1^79^), April 17–23, 2018 (FAROFA2^80^) and April 15–22, 2019 (FAROFA3^81^). The first survey was conducted during the dry season (August to January) while the two others during the rainy season (March to July). Data were collected during daytime while prospecting over the continental shelf, shelf-break and near offshore area (Fig. 1). Video annotations and a degraded resolution of acoustic raw data are published in SEANOE open source platform^82,83^.

### Video data

To identify species and bottom habitat characteristics, four different optical systems were deployed: (i) a towed video camera; (ii) a video camera fixed on the transducer support close to the surface; (iii) a video camera deployed vertically; and (iv) a remotely operated vehicle (ROV) (see Supplementary Fig. S1 online). The towed video camera and the video camera fixed on the transducer were used to capture videos along transects. Both provided a view of the water column and allowed for substratum identification in shallow water. The towed video was set on a downrigger to deepen the camera, which looked downwards and dragged with a 15 m long rope at a vessel speed of 1.5 m s^−1^. The videos captured from the camera fixed on the transducer support were especially useful in very shallow waters. Vertical videos and ROV were deployed during stations whose location was driven by observations of important quantity of fish on the echogram with the purpose of species identification (Fig. 1). Vertical videos were made using a camera fitted on a fishing line. The ROV was operated from the vessel with live stream video.

Towed, fixed and vertical videos were captured with a GoPro TM Hero3+ operating in HD at 1080 p and 30 frames per second during FAROFA 1 and 2 and 60 frames per second during FAROFA 3. ROV videos were performed using a Blue Robotics BlueROV2 system operating in HD at 1080 p and 30 frames per second. To synchronize acoustic and video observations form the towed video, a delay of 9.7 s was subtracted to the video time to adjust with the echosounder time. Each video was annotated using the Solomon Coder software^85^ to identify and enumerate observed species and sediment characteristics classed over nine types (Table 1 and see Supplementary Table S1 online). For the species censuses, we used the maximum number of individuals (MaxN) of a given species present in a single video frame^86^. In video stations, the MaxN was directly used on each frame to avoid double counting of individuals. In video transects, the MaxN over 3 s of record was used. To estimate the abundance of each taxon observed by video, we used the sum of the maximum values of the MaxN of each video (TMaxN).

**Table 1:** Sediment composition description and code.

Composition	Code
Sand	Sa
Large rock + algae	LrAl
Sand + unknown	SaUn
Sand + algae	SaAl
Sand + stone + algae	SaStAl
Sand + large rock + algae	SaLrAl
Sand + rhodolith + algae	SaRhAl
Sand + coral + rhodolith + algae	SaCoRhAl
Sand + stone + coral + rhodolith	SaStCoRhAl

### Acoustic data

Acoustic data were collected continuously throughout the survey with two SIMRAD EK80 echosounders connected to two 7° split beam transducers centred on the frequencies of 70 and 200 kHz and operated simultaneously in narrow band (continuous wave) transmission. Transducers were attached with a stainless-steel pole to the port side of a 10-m-long sport fishing boat. The ping rate was set to ‘maximum’ for a maximum acquisition range of 100 m (over the continental shelf) and to 1 ping s^−1^ off the continental shelf, where the maximum acquisition range was set to 400 m. Vessel speed was ~ 2.5 m s^−1^ during acquisition of acoustic data and pulse duration was set at 1.024 s. Acoustic data were converted to HAC files using Hermes software^87,88^. Processing was completed using the Matecho tool^89^ and Movies3D software^88^. Details on acquisition and calibration parameters as well as on acoustic pre-processing steps from data acquisition i.e., data conversion, bottom detection, filtering and manual cleaning are available in Salvetat et al.^90^.

#### Acoustic data processing

To discriminate fish echoes from other organisms, multifrequency approaches, generally rely on the property that swimbladder-bearing fish have, well beyond the resonance of their swimbladder, high and homogenous backscattering response to frequency^91^. To discriminate between scatters attributed to fish (fish-like) and those originated by other organisms (no-fish), e.g. gelatinous and crustaceans, we developed an approach based on thresholds on (i) volume backscattering strength Sv (Sv, in dB re 1 m^−1^; see^92^ for acoustic definitions), (ii) the bi-frequency sum of Sv, and (iii) the variance of Sv. See Supplementary methods and Supplementary Figs. S2 and S3 online for a detailed description of the methodology. To study the horizontal distribution of fish-like and no-fish echoes, we used the Nautical Area Scattering Coefficient (NASC or s_A_, in m^2^ nm^−2^)^92^, an index of acoustic biomass, for each ping integrated over the water column. Since fish-like and no-fish data were highly correlated at the two frequencies, only the 70 kHz echograms were used for further analyses.

#### Combining surveys

**Figure 2:**
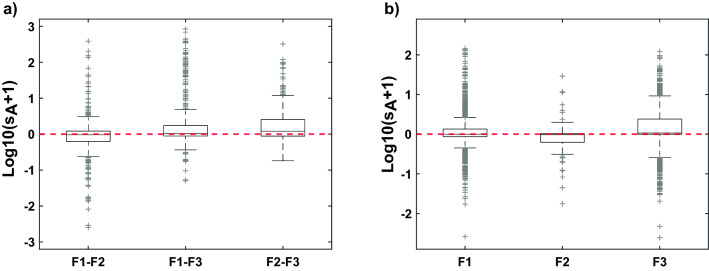
Difference between the mean log10(s_A_ + 1) of different surveys in the selected pixels of 100 m, (**a**) between surveys, and (**b**) within a given survey. s_A_ in m^2^ nm^−2^; F1, F2, F3: FAROFA 1, 2 and 3, respectively.

The three FAROFA surveys were conducted at different seasons and years. To determine whether the surveys could be combined to provide a more comprehensive picture of fish distribution, we verified if, locally, s_A_ values were sufficiently similar between surveys with regards to the natural variability observed within surveys (see Supplementary Fig. S4 online). Punctual comparisons were not possible given that observations of the different surveys were not located at the same geographical points. We thus selected the pixels containing observations from different surveys at a pixel sizes of 100 m (Fig. 2). The inter-survey comparison was based on the difference between the mean log10(s_A_ + 1) of different surveys in the selected pixels. Meanwhile, the intra-survey variability was computed by selecting records at least one hour apart within the same grid cell. The inter-survey differences of fish-like log10(s_A_ + 1) were centred on 0 (Fig. 2a) whatever the pair of surveys considered or the pixel size, and were comparable to intra-survey ones. Based on these results indicating a strong spatial stability in the horizontal fish-like log10(s_A_ + 1) distribution around FNA over years and seasons, we combined the data from the three surveys to provide a comprehensive spatial coverage.

### Spatial distribution

**Figure 3:**
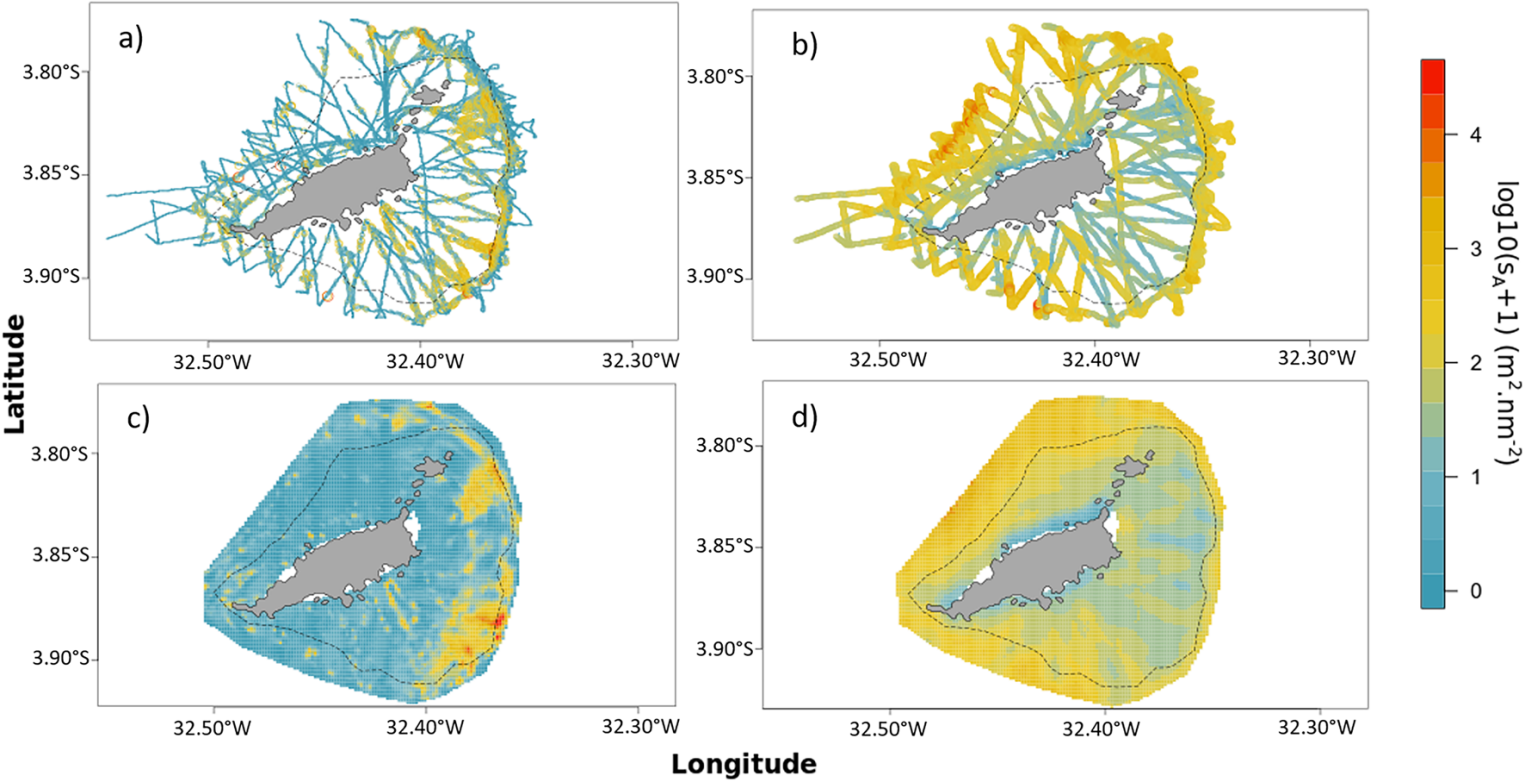
Horizontal distribution of the log10(s_A_ + 1) of fish-like (**a**) and no-fish (**b**) echoes along transects. Geostatistical interpolation of fish-like (**c**) and no-fish (**d**) echoes. The black dashed lines depict the 50 m isobath. Maps were created by the authors using R (https://www.R-project.org/) and RGeostats package^112^.

To interpolate the horizontal distribution of fish-like and no-fish acoustic biomass outside acoustics transects around FNA, we applied a tailored geostatistical approach. Spatial interpolation was adapted to face the fact that the domain area was elliptical with radial transects (Fig. 3a,b). The geographic reference system was thus irrelevant to describe the orientation between observations. For instance, North–South did not mean the same thing in different parts of the survey area. The relevant orientations to consider were rather parallel or perpendicular to the coast, which required projecting the data in a system conformal to these two main orientations.

To unfold the sampling area, we covered the domain by a series of trapezes that were then aligned and resized one by one so that the distance perpendicular to the coast ranged from 0 (coast line) to 1 (offshore border of the trapezes), and the distance parallel to the coast ranged from 0 (beginning of the first trapeze chosen conventionally) to the sum of the length of the bases of the trapezes (Supplementary Fig. S5 online). This projection was bijective so that we could move back and forth between the geographical space and the unfolded space. In particular, the s_A_ and the kriging grid cells whose coordinates were defined in the geographical space were projected in the unfold space to compute their variogram and their kriging values. To avoid border effects at the edge of the unfolded system, the starting and ending trapezes were duplicated prior to kriging. So, the interpolation of the left side of the first trapeze was made taking into account the data of the last trapeze also. Interpolations were performed over regular cells of 55 m longitude × 44 m latitude.

### Fish assemblages

Despite the simultaneous acquisition of acoustic and video, except for two triggerfish species, it was not possible to allocate each fish-like echo to a given species. However, consistent fish assemblages with characteristic echotypes were observed on echograms (Table 2). To attribute each fish-like acoustic scatter to an assemblage, we labelled all fish-like echoes. Label assignment, hereafter called “labelling”, was based on video observation and the presence of characteristic structures in echograms. Video footages made it possible to identify the species observed simultaneously by the cameras and the echosounder. This experience was then used to label the echograms not monitored by videos. For the three cruises, 70 kHz fish-like echograms were labelled manually by the same operator using the software Matecho^89^, which allows drawing polygons to encompass scatters corresponding to a given assemblage. All fish-like echoes inside a polygon were allocated to a given assemblage. In total ten assemblages were defined (Table 2). In addition, two species, the black triggerfish *Melichthys niger* and the ocean triggerfish *Canthidermis sufflamen* could be identified on echograms due to their characteristic shoal shape. The black triggerfish forms large loose shoals occupying the whole water column distributed over the shelf from 6 to 40 m depth exhibiting different body orientations. Ocean triggerfish form smaller looser shoal generally found on deeper depth (~ 17 to 70 m) close to the shelf-break. The fish-like s_A_ of each label corresponding to the 10 assemblages and the two triggerfish species was echointegrated over the water column by 25 m-long elementary sampling distance unit (ESDU).

**Table 2:** Description of the fish assemblages and two fish species defined from echotypes (surrounded by a blue dashed line).

Label name	Characteristics	Species observed in video	Other potential species	Example of echogram
Bottom small fish school	Fish school laying on the bottom. The corresponding fish species are hardly visible on video footage since fish quickly hide in the sediment	*Thalassoma noronhanum* *Halichoeres radiatus*	*Xyrichtys martinicensis* (1) *Heteroconger camelopardalis* (1) *Halichoeres dimidiatus* (2) *Xyrichtys incandescens* (3)	
Bottom weak fish detection	Thin layer of benthic fish	*Gobiidae *sp. *Bothidae *sp.	Crypto-benthic species *Entomacrodus vomerinus* (3) *Ophioblennius atlanticus* (3) *Scartella cristata* (3) *Bathygobius soporator* (3) *Coryphopterus glaucofraenum* (3) *Gnatholepis thompsoni* (3) *Lythrypnus *sp. (3) *Priolepsis dawsoni* (3)	
*C. sufflamen*	Loose shoal of fish, distributed close to the shelf-break	*Canthidermis sufflamen*		
Individual demersal fish	Individual fish on the bottom or in the water column over the shelf	*Lutjanus jocu* *Sphyraena barracuda* *Caranx lugubris* *Caranx latus*		
Loose school	Loose school of unidentified fish over the shelf			
*M. niger*	Large loose shoal with fish exhibiting different body orientations, distributed over the shelf	*Melichthys niger*		
Mix reef fish	Fish schools and shoals over complex bottom structure formed by coral or rocky reefs	*Abudefduf saxatilis* *Chromis multilineata* *Melichthys niger* *Sparisoma amplum* *Acanthurus coeruleus, A. chirurgus* *Stegastes rocasensis* *Cephalopholis fulva* *Kyphosus sectatrix* *Cantherhines macrocerus* *Lutjanus jocu* *Thalassoma noronhanum* *Haemulon chrysargyreum* *Balistes vetula* *Paranthias furcifer* *Pseudupeneus maculatus* *Holocentrus adscensionis*	*Sparisoma axillare *(2) *S. frondosum *(2),* S. radians *(4) *Anisotremus surinamensis *(3) *Haemulon parra *(3) *Haemulon chrysargyreum *(3) *Rypticus saponaceus *(3) *Dermatolepis inermis *(3) *Mycteroperca bonaci *(3) *Epinephelus itajara *(3) *Pomacanthus paru *(3) *Holacanthus ciliaris *(3) *Myripristis jacobus *(3) *Mulloidichthys martinicus *(3) *Pempheris schomburgki *(3) *Centropyge aurantonotus* *Chaetodon striatus *(3)*, C. ocellatus *(3)	
Sand fish	Fish school over flat sand bottom	*Dactylopterus volitans* *Hypanus americanus* *Aetobatus narinari*		
Shelf-break large fish	Individual large fish in the water column over the shelf-break	*Sphyraena barracuda* *Caranx lugubris* *Seriola dumerili* *Elagatis bipinnulata* *Thunnus *spp. *Caranx *spp. *Carcharhinus falciformis*		
Shelf-break school	Demersal fish school associated to the shelf-break		Mix of reef fish *Paranthias furcifer* *Kyphosus sectatrix* *Caranx lugubris* *Mycteroperca *spp*. *(1) *Menophorus dubius *(1) *Ginglymostoma cirratum *(1) *Prognathodes guyanensis *(1) Lujanidae	
Small pelagics school	Fish in dense large school characteristic of small pelagic fish schools	*Decapterus macarellus* *Harengula *sp.	*Harengula jaguana* (5) *Harengula clupeola* (3)	
Small pelagics and predators	Loose shoal of small pelagic fish in interaction with predators	*Sphyraena barracuda* *Caranx lugubris* *Seriola dumerili* *Elagatis bipinnulata* *Thunnus *spp. *Caranx *spp. *Caranx crysos* *Carcharhinus falciformis*		

^(1)^Barros (2020)^93^.

^(2)^Sazima et al. (2005)^94^.

^(3)^Soto (2001)^95^.

^(4)^Krajewski and Floeter (2011)^96^.

^(5)^Sazima et al. (2006)^97^.

### Black triggerfish biomass estimation

The black triggerfish was particularly abundant in observations. The availability of target strength measurement for this species^90^ opened the field for estimating its biomass. To account for the strong dissymmetry of s_A_ histograms, we used a non-linear geostatistical approach^98–100^. Observations were reduced to 5 classes of acoustic biomass, i.e. null, small, medium, large and very large densities, corresponding to the classes 0, ]0–33%], [33–66%], [66–95%], [95–100%], respectively. Each interval was coded by an indicator variable, the first one being nothing but the presence/absence. This coding translated the univariate approach of s_A_ into a multivariate approach (five disjunctive indicator variables, that reduced to four as they sum to one). This became a real issue given the very large number of s_A_ data available. To solve this problem, the five spatially mutually correlated indicators variables were transformed into five factors called Min–max Autocorrelation Factors—MAF^101^. These factors are linear combinations of the input georeferenced variables, and are uncorrelated at null and at short distances. Assuming that MAFs were also uncorrelated for larger geographical distances, allowed using them independently from the others. In this context, we performed the global estimation of each of the five MAFs over the study polygon by global kriging^99^ and then recombined them to get the kriging estimates of the mean overall s_A_ together with its estimation variance^102^.

Using all black triggerfish labels, we delineated its area of main presence, concentrated in the east side of FNA. In this area, based on the estimation of the mean overall acoustic value described above, we estimated the biomass by Eq. (1):

1 $$Biomass = mean\;overall\; s_{A} \times {\text{Surface}} \times {\text{W}}/\left( {1852^{2} \times { }4{\pi } \times 10^{{\frac{TS}{{10}}}} } \right)$$

where the *Surface* corresponds to the total surface of the delineated area (in m^2^), *W* is the triggerfish mean weight estimated at 485 g for 27.8 cm long black triggerfish and *TS* is the target strength of the black triggerfish at 70 kHz (*TS* = − 39.3 dB for 27.8 cm long black triggerfish, the mean size during the surveys^90^).

### Environmental drivers

We investigated the relationships between the fish-like and no-fish acoustic biomasses to a series of categorical environmental variables:

- Wind/current exposure: FNA was categorised in two sides, leeward and windward, according to the exposition to main winds and currents. Indeed, FNA is under the trade wind regime and washed by the central branch of the South Equatorial Current (cSEC) that both flow from east to west^103,104^.
- Depth strata: data were classified in two euphotic (upper euphotic: 0–20 m; lower euphotic: 20–40 m) and three mesophotic (upper mesophotic: 40–60 m; mid-mesophotic: 60–80 m and lower mesophotic: 80–100 m) depth strata using the acoustically-detected bottom-depth.
- MPA: data were compared inside and outside the PARNAMAR in the same depth range (0–50 m). The area outside the MPA belongs to the multiple use Environmental Protection Area (EPA).
- Sediment type: the nine sediment types extracted from video observation (Table 1).

We used both univariate and multivariate statistics to relate the distribution of the acoustic biomasses log10(s_A_ + 1) of fish-like and no-fish data as well as the acoustic biomasses of the ten assemblages plus the two triggerfish species to the environmental factors. To seek for significant differences in acoustic biomass according to each environmental factor, we used a non-parametric Kruskal Wallis test followed by pairwise Wilcoxon tests since the distribution of the data did not follow a normal distribution.

Classification And Regression Trees (CART^105^) were used to explore the relationships between the fish-like acoustic biomass and the fish assemblages (plus the two triggerfish species), and environmental variables. In the first case, we used the rpart package^106^ while we used the diet package^107^ in the second case. The diet package, originally designed to study diet composition, allows a non-parametrical exploratory and predictive approach for identifying complex relationships between environmental variables and assemblages composition. Classification tree using the diet package uses a bootstrap technique similar to Breiman^108^ and Kuhnert et al.^109^. The diet package also allows visualizing the bagged predictions by mapping the predictions^110^. Both trees were pruned to the smallest cross-validated relative error^105^.

In addition to the categorical environmental factors, we added two continuous explicative variables, the no-fish acoustic biomass and the bottom local slope (absolute value of the difference between the first and the last depth in a given ESDU relative to the length of the ESDU expressed in %). All statistical analyses were performed with R^111^.

All statistical analyses were performed twice, including and not the sediment types. Indeed, the sediment types were extracted from video observation and were thus not available for all ESDU but mostly restricted to the shallow areas where videos observations are available and the sediment observable.

## Results

### Video data

In total, 49h51 of video footage were acquired. Complete information on species identification and sediment classification are available at 10.17882/76019. Video footage allowed the identification of 47 taxa (Table 3) from 29 families including one turtle (*Chelonia mydas*), one dolphin (*Stenella longirostris*), six elasmobranchs including four sharks (*Carcharhinus falciformis, C. perezi, Sphyrna lewini* and *Ginglymostoma cirratum*) and two stingrays (*Aetobatus narinari* and *Hypanus americanus*). We identified thirty-four osteichthyes fish species at specie level, four at gender level (*Harengula *sp.,* Seriola *spp., *Caranx *spp., *Thunnus *spp.) and three at family level (*Gobidae *sp., *Bothidae *sp., *Ostraciidae *sp.). For 12 species, only a single individual was recorded. 49,189 fish were recorded using the TMaxN. The most abundant specie, *Harengula *sp. (TmaxN 23529, 47.8%) was observed forming large schools in 2 videos, while the second most abundant species, the black triggerfish *Melichthys niger* (TMaxN 21416, 43.5%) was the fish observed in more videos (48). After those two species, the relative abundance falls with only three species with abundance above 1% of the total abundance: the sergeant major *Abudefduf saxatilis* (TMaxN 1495, 3%), the brown chromis *Chromis multilineata* (TmaxN 636, 1.3%), the ocean triggerfish *Canthidermis sufflamen* (TMaxN 540, 1.1%). After *M. niger* that occurred in 48 videos the species observed in more videos were the barracuda *Sphyraena barracuda* (45), the oceanic triggerfish *Canthidermis sufflamen* (39), the black jack *Caranx lugubris* (22), the blue runner *Caranx crysos* (13), the sergeant major *A. saxatilis* (12), and the dog snapper *Lutjanus jocu* (12).

**Table 3:** List of species observed in video footages. *TMaxN* sum of the maximum number of individuals of a given species present in a single video frame, *TV* towed video, *TS* transducer support, *VP* vertical profile, *RV* ROV. The numbers in parenthesis associated to video types indicates the number of videos in which each species was observed.

Class	Order	Family	Species	TMaxN	Video type
Actinopterygii	Beryciformes	Holocentridae	*Holocentrus adscensionis*	1	TV(1)
	Clupeiformes	Clupeidae	*Harengula *spp.	23,529	TS(2)
	Perciformes	Acanthuridae	*Acanthurus coeruleus*	8	TV(2),VP(1)
			*Acanthurus chirurgus*	21	TS(1)
		Carangidae	*Caranx crysos*	189	TV(7),VP(5),RV(1)
			*Caranx latus*	53	TV(6)
			*Caranx lugubris*	88	TV(10),TS(1),VP(10),RV(1)
			*Caranx ruber*	51	VP(3),RV(2)
			*Caranx *spp.	57	TV(2),RV(1)
			*Decapterus macarellus*	266	VP(1)
			*Elagatis bipinnulata*	124	TV(3), VP(6)
			*Seriola *spp.	7	VP(5)
		Gobiidae	*Gobiidae *sp.	1	TV(1)
		Haemulidae	*Haemulon chrysargyreum*	149	TV(1)
		ITSiophoridae	*Makaira nigricans*	1	TV(1)
		Kyphosidae	*Kyphosus sectatrix*	182	TV(3), VP(1)
		Labridae	*Halichoeres radiatus*	1	TV(1)
			*Thalassoma noronhanum*	43	TV(2), VP(1)
		Lutjanidae	*Lutjanus jocu*	25	TV(6), VP(5), RV(1)
		Malacanthidae	*Malacanthus plumieri*	4	TV(2)
		Mullidae	*Pseudupeneus maculatus*	1	TV(1)
		Pomacentridae	*Abudefduf saxatilis*	1495	TS(3), TV(5), VP(4)
			*Chromis multilineata*	636	TS(2), TV(4),VP(2)
			*TSegaTSes rocasensis*	17	TV(1), VP(1)
		Serranidae	*Cephalopholis fulva*	18	TV(1), VP(2)
			*Paranthias furcifer*	57	TV(2)
	Pleuronectiformes	Bothidae	*Bothidae *sp.	1	TV(1)
	Scombriformes	Scaridae	*Sparisoma amplum*	3	TV(1), VP(1)
		Scombridae	*Acanthocybium solandri*	11	TV(2),RV(1)
			*Thunnus albacares*	1	RV(1)
			*Thunnus *spp.	35	VP(1)
		Sphyraenidae	*Sphyraena barracuda*	106	TV(18), VP(23), RV(4)
	Scorpaeniformes	Dactylopteridae	*Dactylopterus volitans*	2	VP(2)
	Tetraodontiformes	BaliTSidae	*BaliTSes vetula*	2	TV(1)
			*Canthidermis sufflamen*	540	TV(18), VP(20), RV(1)
			*Melichthys niger*	21,416	TS(1),TV(26), VP(19), RV(2)
		Monacanthidae	*Aluterus scriptus*	21	VP(8)
			*Cantherhines macrocerus*	9	TV(4), RV(1)
		OTSraciidae	*Lactophrys trigonus*	1	VP(1)
			*OTSraciidae *sp*.*	1	TV(1)
Chondrichthyes	Carcharhiniformes	Carcharhinidae	*Carcharhinus falciformis*	1	VP(1)
			*Carcharhinus perezi*	1	VP(1)
		Sphyrnidae	*Sphyrna lewini*	1	RV(1)
	Myliobatiformes	Dasyatidae	*Hypanus americanus*	2	TV(1), VP(1)
		Myliobatidae	*Aetobatus narinari*	2	TV(1)
	Orectolobiformes	GinglymoTSomatidae	*GinglymoTSoma cirratum*	2	TV(2)
Mammalia	Cetacea	Delphinidae	*TSenella longiroTSris*	1	TS(1)
Reptilia	TeTSudinata	Cheloniidae	*Chelonia mydas*	3	TS(2)

Beside the fish species, we observed gelatinous (salps, siphonophores and ctenophores), fish larvae (including leptocephalus) and fragments of pelagic algae. These, and likely crustaceans that could not be observed in videos, are the main components of the ‘no-fish’ acoustic data.

### Fish-like and no fish acoustic biomass

The geostatistical interpolation of fish-like s_A_ reveals a heterogeneous distribution with the presence of several hotspots, mostly on the windward (east) side of FNA, in the vicinity of the shelf-break (Fig. 3c). The lowest fish-like acoustic biomass was observed on the north-western side, in particular at the mid-shelf. The pattern was different for the no-fish acoustic biomass that was concentrated off the shelf-break with a main aggregation on the leeside (Fig. 3d).

**Figure 4:**
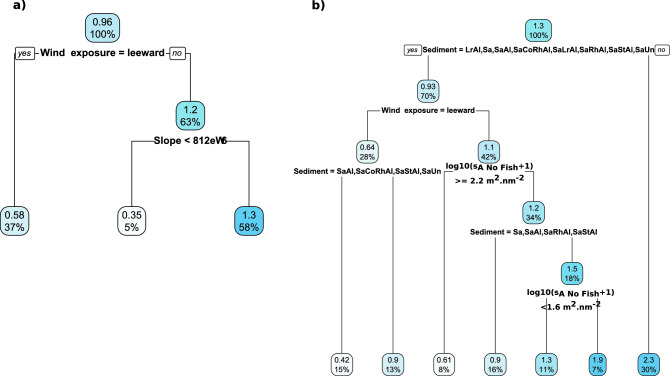
Regression tree on values of the fish-like acoustic biomass (log10(s_A_ + 1)) performed on the complete data set (**a**) and the dataset limited to the locations where sediment was observed (**b**). The values inside the each leaf is the mean fish-like acoustic biomass (log10(s_A_ + 1)) and the corresponding percentage of ESDU.

The regression tree relating the fish-like acoustic biomass to the environmental parameters without considering the sediment (Fig. 4a) reveals that the main driving factor is the wind exposure with much higher acoustic biomass per ESDU windward (log10(s_A_ + 1) = 1.2 m^2^ nm^−2^) than leeward (0.58 m^2^ nm^−2^). A second split occurs in the windward side with higher acoustic biomass (1.3 vs. 0.35 m^2^ nm^−2^) when the slope is greater than 0.08% meaning that fish-like acoustic biomass is very low in flat areas. When considering the reduced database (restricted to the neritic zone) containing sediment (Fig. 4b), the first explicative variable is the sediment. The most complex sediment (SaStCoRhAl) encompasses a much higher biomass (2.3 m^2^ nm^−2^) than the others (0.93 m^2^ nm^−2^). For sediments other than SaStCoRhAl, the next splits are wind exposure, sediment types and no-fish biomass with the highest fish-like biomass (1.9 m^2^ nm^−2^) distributed windward where the no-fish acoustic biomass ranges between 2.3 and 1.6 m^2^ nm^−2^ and over the more complex sediments (SaCoRhAl, SaLrAl, LrAl, SaUn).

Univariate analyses provide some additional information (see Supplementary Fig. S6 online). Fish-like and no-fish acoustic biomasses significantly varied according to wind and current exposure, protection levels, sediment types, and bottom depth strata. Indeed fish-like acoustic biomass was significantly lower on the leeward even if some ESDU encompassed very high acoustic biomass (log10(s_A_ + 1) up to 6.8 m^2^ nm^−2^) in presence of small pelagic schools distributed in the upper mesophotic zone (40–60 m) (see “Fish assemblages”). On the opposite, the no-fish acoustic biomass was slightly (but significantly) lower windward (2.1 m^2^ nm^−2^) than leeward (2.2 m^2^ nm^−2^).

The type of sediment encompassing the highest acoustic biomass was by far the most complex one, SaStCoRhAl (mean log10(s_A_ + 1) = 2.16 m^2^ nm^−2^), followed by SaCoRhAl (1.14 m^2^ nm^−2^), SaLrAl (1.11 m^2^ nm^−2^), LrAl (1.06 m^2^ nm^−2^), SaStAl (0.99 m^2^ nm^−2^), SaRhAl (0.95 m^2^ nm^−2^), while the less complex habitats Sa (0.78 m^2^ nm^−2^), SaAl (0.77 m^2^ nm^−2^) and SaUn (0.70 m^2^ nm^−2^), presented the lowest mean acoustic biomass and a strong dominance of zero values. The no-fish acoustic biomass did not present any clear association with the sediment complexity since the higher acoustic biomass was associated to SaUn (mean log10(s_A_ + 1) = 1.94 m^2^ nm^−2^), followed by SaStCoRhAl (1.93 m^2^ nm^−2^), SaCoRhAl (1.86 m^2^ nm^−2^), SaStAl and Sa (1.77 m^2^ nm^−2^), SaAl and SaRhAl (1.66 m^2^ nm^−2^), SaLrAl (1.30 m^2^ nm^−2^), and LrAl (0.69 m^2^ nm^−2^).

Fish-like acoustic biomass was significantly higher (mean log10(s_A_ + 1) = 1.14 m^2^ nm^−2^) in the mid-mesophotic zone (60–80 m) that encompasses the upper edge of the shelf-break than the lower euphotic (1.07 m^2^ nm^−2^), followed by the upper mesophotic (0.92 m^2^ nm^−2^), the upper euphotic (0.87 m^2^ nm^−2^) and the lower mesophotic (0.85 m^2^ nm^−2^). The no-fish acoustic biomass significantly increased with the bottom depth. It was higher in the lower mesophotic strata (mean log10(s_A_ + 1) = 2.49 m^2^ nm^−2^) where dense and strong layers of gelatinous were observed, than in the mid-mesophotic (2.25 m^2^ nm^−2^), followed by the upper mesophotic (2.08 m^2^ nm^−2^), the lower euphotic (1.84 m^2^ nm^−2^), and the upper euphotic zone (1.37 m^2^ nm^−2^).

Finally, fish-like acoustic biomass was significantly higher inside (mean log10(s_A_ + 1) = 1.18 m^2^ nm^−2^) than outside (0.46 m^2^ nm^−2^) the no-take zone. Although less marked, the same trend was observed for the no-fish acoustic biomass (1.88 m^2^ nm^−2^ vs. 1.75 m^2^ nm^−2^).

### Fish assemblages

Fish-like echoes were assigned to ten assemblages and two triggerfish species (Table 2). Video observations allowed a good identification of the species present for most of the groups. However, the composition of four groups (bottom weak fish detection, sand fish, loose school, shelf-break school) could not be fully validated by the videos.

**Figure 5:**
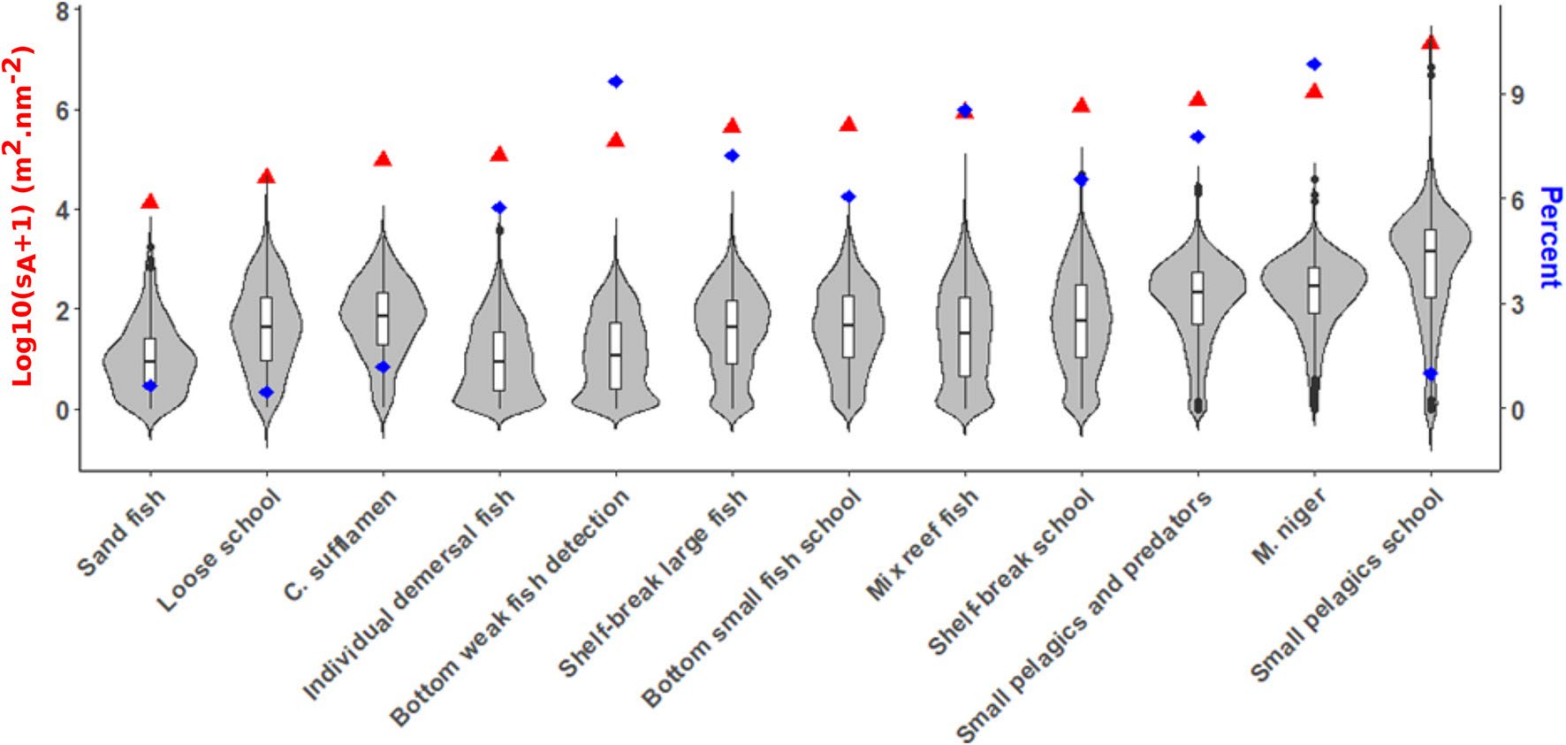
Violin plot containing boxplot representing the median (horizontal line), interquartile range, whiskers and outlying points of the acoustic biomass of individual assemblages (in log10(s_A_ + 1)) by ESDU, their cumulative sum (red triangles) and the percentage of ESDU with presence of each assemblages (blue diamonds).

Small pelagic school presented the highest total acoustic biomass, followed by the black triggerfish (Fig. 5). The percentage of ESDU with presence of a given assemblage also mostly followed the trends in acoustic biomass with some notable exceptions. Small pelagic fish school that encompassed the highest acoustic biomass was only observed in 1% of ESDU (Fig. 5), indicating that they were concentrated within some large schools. On the opposite, *M. niger* was the most frequently observed assemblage (in 9.9% of ESDU) followed by bottom weak fish detection (9.4%) that ranked eighth in terms of total acoustic biomass.

**Figure 6:**
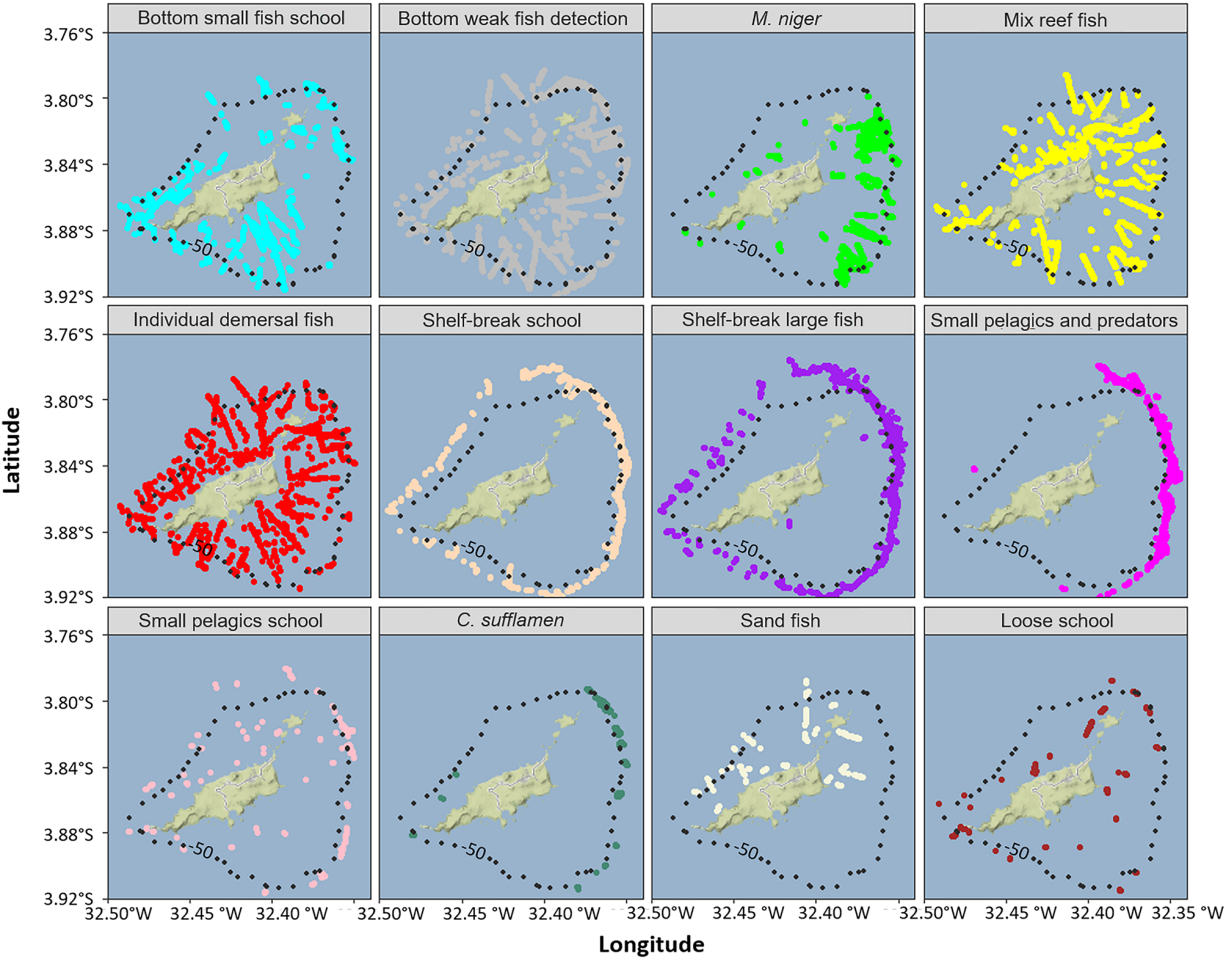
Spatial distribution represented by coloured points of the fish assemblages. The black dotted line depict the 50 m isobath. Maps were created by the authors using R (https://www.R-project.org/) and ggmap^113^.

Fish assemblages presented specific spatial patterns of distribution (Fig. 6). Four assemblages (bottom small fish school, bottom weak fish detection, individual demersal and mix reef fish) presented the most comprehensive distributions over the shelf, all around FNA. The other assemblages associated to the shelf were loose school and sand fish, mostly distributed close to the coast and *M. niger*, mainly distributed on the windward side of FNA. Small pelagic schools were distributed both over the shelf and at the shelf-break. The other groups were mostly associated to the shelf-break, with shelf-break schools and shelf-break large fish distributed all over FNA while small pelagic and predators and *C*. *sufflamen* were mostly distributed on the windward side.

**Figure 7:**
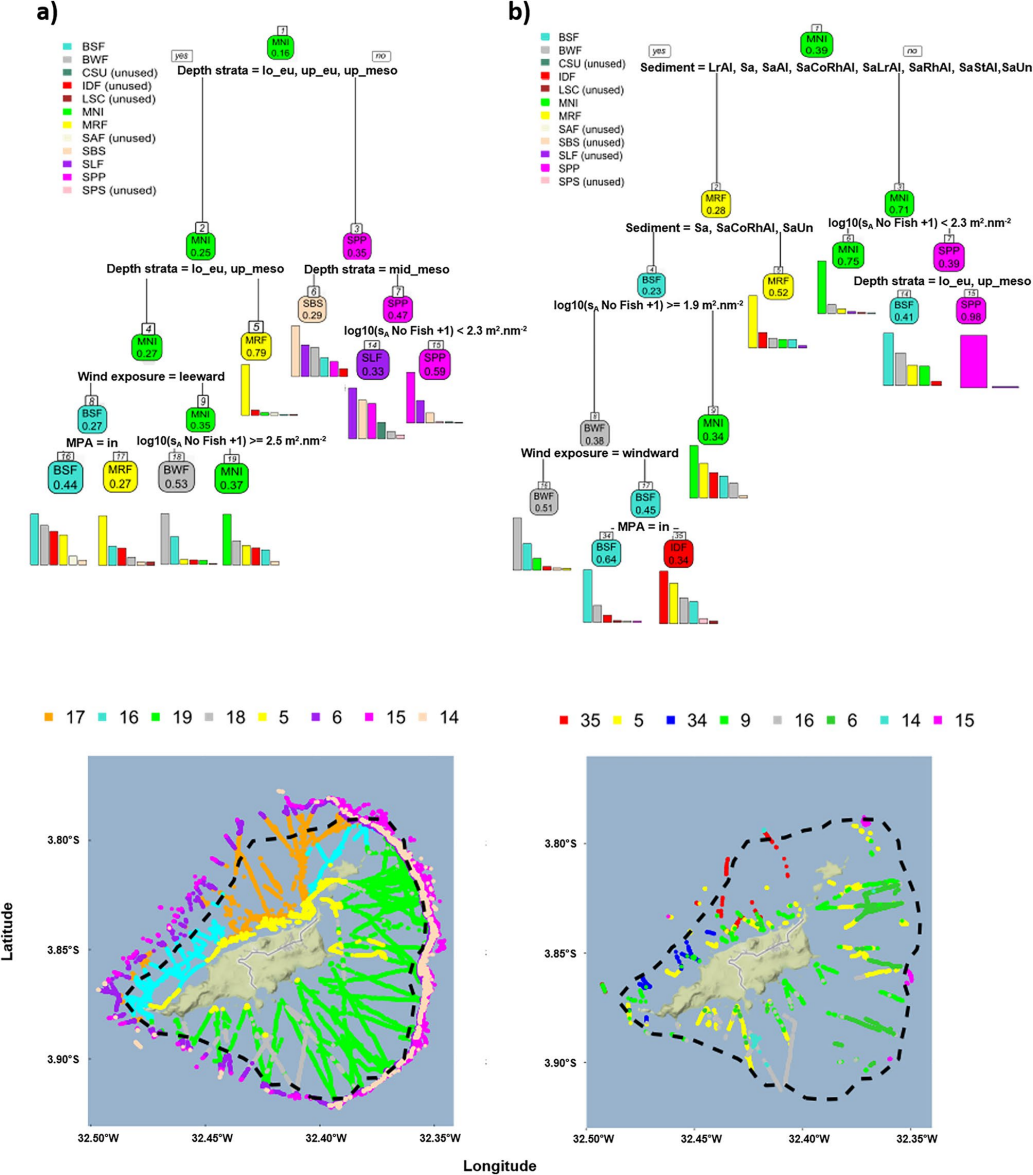
Regression tree (**a**,**b**) and associated prediction map (**c**,**d**) performed on the acoustic biomass (in log10(s_A_ + 1)) of fish assemblages according to the environmental parameters with the complete data set (**a**,**c**) and the dataset limited to the locations with observed sediment (**b**,**d**). The fish assemblages identified at each terminal node are those with the highest proportion composition in percent. The composition in assemblage percent for each terminal node is represented by the histogram beneath it. Covariates used to develop the tree were depth strata (*up_eu* upper euphotic, *lo_eu* lower euphotique, *up_meso* upper mesophotic, *mid-meso* mid-mesophotic, *lo_meso* lower mesophotic), position (wind exposure: windward or leeward), protection level (MPA: in or out) sediment (see Table 1 for sediments codes), fish-like and no-fish acoustic biomass (s_A_). Fish assemblages abbreviations were, *BSF* bottom small fish, BWF: bottom weak detection, IDF: individual demersal fish, MNI: *M. niger*, MRF: mix reef fish, SBS: shelf-break school, SLF: shelf-break large fish, SPP: small pelagics and predators. Trees were made by the authors using diet package^107^. Maps were created by the authors using R (https://www.R-project.org/) and ggmap^113^.

The regression tree relating the fish-like acoustic biomass to the environmental parameters without considering the sediment (Fig. 7a,c) reveals that the main driving factor is depth strata, discriminating between areas shallower (upper and lower euphotic, upper mesophotic) and deeper than 60 m depth (mid and lower mesophotic). Bottom depths shallower than 60 m correspond to the shelf where neritic assemblages dominate: bottom small fish school, mix reef fish, bottom weak fish detection, *M. niger*. Pelagic assemblages logically dominate in deeper areas: small pelagics and predators, shelf-break large fish and shelf-break school. Over the shelf, the next splits of the trees are depth strata, wind exposure, MPA protection and s_A_ no-fish. Mix reef fish constituted 79% of the assemblages on the upper euphotic strata, it was also the dominant group (27%) in the lower euphotic and upper mesophotic strata on the leeward side outside the MPA. *M. niger* dominates (37%) on lower euphotic and upper mesophotic strata on the windward side with low no-fish acoustic biomass. On the pelagic side, shelf-break large fish dominated (29%) on the mid-mesophotic zone 60–80 m and small pelagic and predators (47%) on the lower mesophotic zone 80–100 m. When considering the reduced database containing sediment information (Fig. 7b,d), the first explicative variable was the sediment. On SaStCoRhAl sediment, *M. niger* dominates (73%)*.* For sediments other than SaStCoRhAl, the next splits of the trees are sediment, no-fish acoustic biomass, wind exposure, MPA protection and depth strata. On the sediments LrAl, SaAl, SaRhAl, SaLrAl, SaStAl 52% of the assemblages belong to mix reef fish.

Univariate analyses showed that the percentage of space occupation was substantially higher on the windward side for half of the assemblages (*C. sufflamen,* bottom weak fish detection, shelf-break large fish, shelf- break school, small pelagics and predators, *M. niger*) (Supplementary Fig. S7 online). The highest acoustic biomass of all groups corresponded to small pelagics school in the leeward side, followed by *M. niger* in the windward side and mix reef fish in the leeward side.

All assemblages (Supplementary Fig. S7 online) varied substantially according to the bottom depth strata. The acoustic biomass percent of presence were higher in the upper and lower euphotic strata for mix fish and *M. niger*, respectively. The acoustic biomass and percent of presence of demersal assemblages, sand fish and mix reef fish, decreased with depth. The opposite occurred for pelagic groups shelf-break large fish, shelf-break school, small pelagics and predator, *C. sufflamen* that peaked at mid and lower mesophotic depths, except for small pelagic school that presented highest acoustic biomass in the upper mesophotic zone (40–60 m). Bottom weak fish detection and bottom small fish school, individual demersal fish were distributed and presented higher acoustic biomass either on the lower euphotic or upper mesophotic.

Sediment identification was only possible on shallow water and represented a small portion of the data. In this dataset, some assemblages presented clear association with one sediment (Supplementary Fig. S7 online). In particular mix reef fish were strongly related with SaLrAl, *M. niger* with SaStCoRhAl and bottom small fish school with Sa. Mix reef fish and *M. niger,* although showing a higher occurrence on a particular substrate, were the two only groups that appeared on all sediment types.

**Figure 8:**
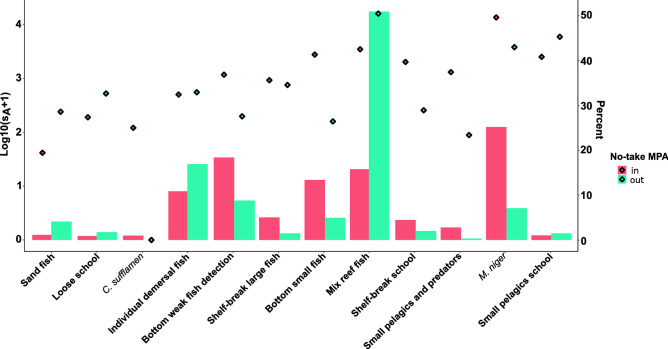
Barplot representing the percentage of ESDU with presence of each fish assemblage according to its position regarding the no-take zone and the mean acoustic biomass of each group (log10(s_A_ + 1)) per ESDU (i.e. the total fish acoustic biomass normalised by the number of ESDU inside and outside the no-take zone) (diamonds).

The no-take MPA effect was clear on fish distribution as six assemblages (bottom weak fish detection, shelf-break large fish, bottom small fish school, shelf-break school, small pelagics and predators, *M. niger*) were more present and had higher acoustic biomass inside the MPA (Fig. 8). *C. sufflamen* was virtually absent outside the no-take MPA. The five remaining assemblages (sand fish, loose school, individual demersal fish, mix reef fish and small pelagic school), were all more present and presented higher acoustic biomass outside than inside the no-take MPA.

### Black triggerfish biomass

**Figure 9:**
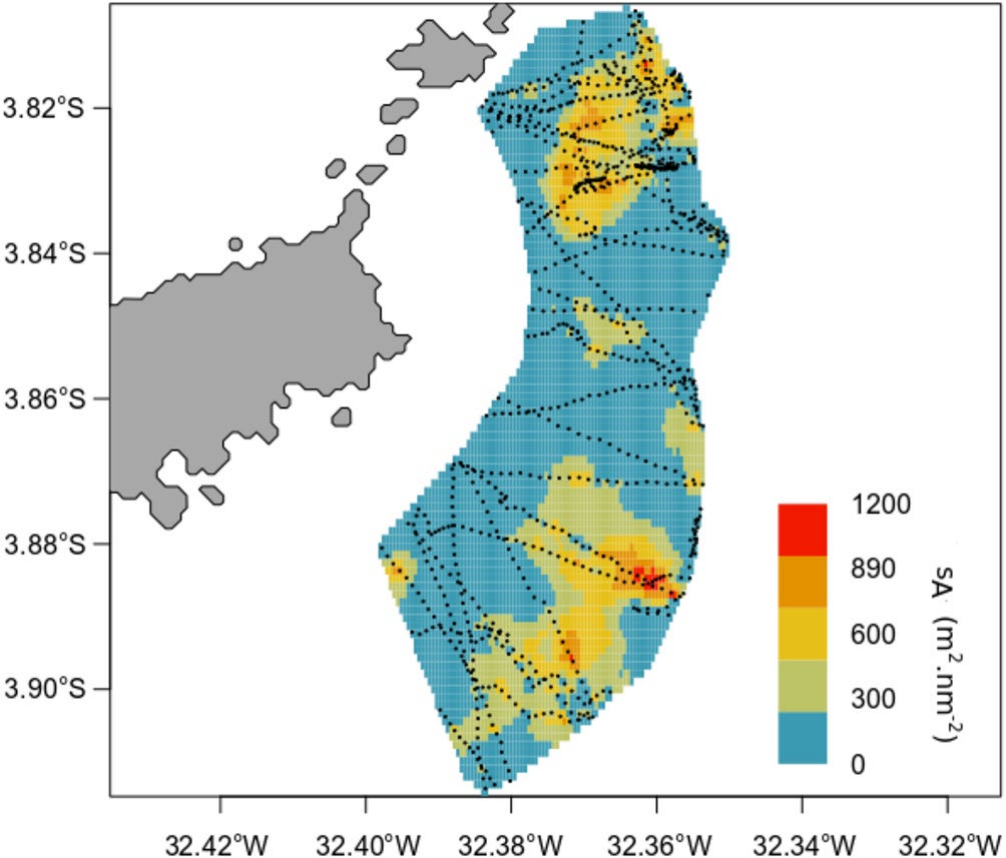
Interpolated distribution of the black triggerfish distributed in 5 classes of values of s_A_. The black dashed lines represent the acoustic transects. Map was created by the authors using R (https://www.R-project.org/) and RGeostats package^112^. Created with Adobe Illustrator software (https://www.adobe.com) by graphic designer Pierre Lopez.

The black triggerfish, the second group in terms of fish acoustic biomass, was mostly concentrated in the East side of FNA. In this area (Fig. 9), its actual biomass was estimated to 700 tonnes (19 g m^−2^) with an estimated CV of 40%. Its distribution inside the area was heterogeneous and organised in patches.

## Discussion

By combining acoustic and video observations, we provided for the first time, a comprehensive vision of tropical fish distribution from the near-shore line to off the shelf-break with a description of (i) fish diversity, (ii) horizontal fish-like and no-fish distribution, and (iii) a focus on the black triggerfish. The data gathered also provided important evidence to (iv) revisit the Island Mass Effect (IME), and (v) give some insights for Marine Spatial Planning (MSP).

### Fish diversity

In about 50 h of video footage, we recorded 47 fish species of 29 families (Table 3). This is only a small fraction of the ichthyofauna of FNA that, with a total of 250 species and 77 families, harbours the greatest richness of marine fish among the oceanic islands of the South Atlantic^96,114^. Indeed our objective was not to perform an exhaustive description of the fish diversity but to capture the fish composing the bulk of the biomass. Still, compared with other visual census techniques we observed a similar number of families (27–28^96,115,116^) but much fewer species (50–66)^96,115,116^. Only, Soto^95^ depicted much more families (68) and species (167) but his inventory included pelagic species combining visual census, fisheries surveys and literature records. The most diverse families (Serranidae, Labridae and Pomacentridae) are underrepresented in our study since we focused on pelagic and demersal species that can be assessed with acoustics and did not put efforts on filming benthonic and cryptobenthic communities.

In our study, two species, the tropical sardine *Harengula *sp. and the black triggerfish *M. niger* accounted for more than 90% of the fish recorded. This confirm the fact that the biodiversity in FNA is represented by few very abundant species^117^. *M. niger* was the second most abundant and the most often observed species. Such results differ from other studies in FNA. Indeed, most studies^96,115,118^ report *Thalassoma noronhanum*, *Haemulon chrysargyreum* and *Stegastes rocasensis* as dominant species. The difference between our and other studies is likely due to our extensive depth coverage compared with other that mostly focused on shallow (< 20 m) waters. Only Schmid et al.^116^ observed a dominance of *M. niger* but their study was performed using baited remote underwater video stations and black triggerfish are voracious bold species^119^. Even if far form exhaustive, our video records likely provide a robust picture of the main pelagic and demersal species present in FNA and a robust information to complement and identify acoustic observations.

Fish echotypes are known to provide a heuristic description of fish species, assemblages or communities^120,121^. Combining video observations with the scrutinising of acoustic echograms allowed for the identification of consistent fish assemblages (Table 2). We acknowledge that, in some cases, some fish echotraces may not have been correctly assigned to the proper fish assemblage. However, since these assignations were sustained by ~ 50 h of video footage we are confident that potential mis-assignation should not have significantly impacted our results.

### Comprehensive tropical fish distribution

The algorithm we applied on acoustic data allowed discriminating between fish-like and no-fish echograms. The access to simultaneous video observation and the care taken to validate the algorithm with these images makes us confident that the fish-like echograms do indeed correspond to fish.

The strong stability in acoustic fish biomass distribution between FAROFA surveys (Fig. 2), through years and seasons that allowed merging the data from the three surveys, suggests a bottom-up structuration of fish assemblage. Most fish observed by video and acoustics are demersal and pelagic. Demersal fish are classically associated with typical habitat in terms of sediment, benthic composition, structural complexity or depth^24,122–124^ (see also “Revisiting the Island mass effect (IME)”), which may explain site-fidelity. In the same way, except for *Harengula *sp., the pelagic fish species were concentrated at the shelf-break, a known hotspot for pelagic fish^75^. Seasonal variations (e.g. rainy vs. dry season) do not imply significant environmental changes. Indeed, the seasonal variation gradient sea surface temperature is minimal (varying from 26.5 to 28 °C)^34^ and does not seem to significantly impact the distribution of the fish as observed by acoustic. A similar result with no change regarding the season was found in fish predator diet^125^.

The comprehensive spatial coverage we achieved allows providing the first map of fish acoustic biomass around FNA (Fig. 3). Such a picture cannot be completed with classic methods based on visual census or fishing operations (in systems where fishing activity is allowed). More generally, this is the first comprehensive high-resolution map of fish distribution of a tropical system from the near-shore to the shelf-break. To our knowledge at least one example of map of tropical fish biomass was produced from acoustic data covering a fraction of the U.S. Virgin Islands^126^. However, this study did not use simultaneous video observation limiting the skills of species identification. By combining acoustics and video, we provide the spatial distribution of all acoustically detected fish (Fig. 3) but also of a variety of fish assemblages (Fig. 6). Our results reveal a strong heterogeneity in the distribution of fish acoustic biomass with the presence of hotspots. This reinforces the fact that punctual observations may miss hotspots in comparison with our extensive continuous sampling. The comprehensive maps we provide have several benefits since they can help defining areas for further sampling strategies, and are key elements for management in particular for implementing MSP^6,38,77^.

### Black triggerfish: a key tropical player

In phase with video observations, the black triggerfish was the second most important species in terms of acoustic biomass and the one occupying most space. In its main zone of distribution, we estimated the black triggerfish biomass to 700 tonnes (i.e. 19 g m^−2^). *M. niger* is one of the very few reef fish with a circumtropical distribution^127^. It is known to form large shoals of more than one hundred individuals and has been reported to exhibit high densities around remote oceanic islands such as Ascension Island^128,129^, Clipperton Atoll^130^, Trinidad Island^131^, Johnson atoll and Porto Rico^127^ or St Peter and St Paul’s Rocks^132,133^. *M. niger* thrives at colonizing and maintaining high population levels at remote location^127^. This is probably due to its long pelagic phase that enables its settlement in remote location and its high plasticity in resource use. Indeed, *M. niger* broad omnivory gives him the potential to forage opportunistically on a variety of prey, including pelagic algae remains or dolphin vomit and faeces^97^. In addition, isolated oceanic Islands such as FNA, are impoverished and the functional richness is low^96,134,135^. *M. niger* has the ability to take advantage of an empty niche as demonstrated by Mendes et al.^119^ at St Peter and St Paul Archipelago where it endorses the functional role of opportunistic grazing herbivore. FNA lacks of large herbivore that are represented by few small Scaridae of the gender *Sparisoma*^136^ and roving herbivore represented by few Kyphosidae and Acanthuridae. In our study we observed one species of parrotfish *Sparisoma amplum* (3 individuals in 2 videos), one species of sea chub *Kyphosus sectatrix* (183 individuals in 4 videos) and one surgeon fish *Acanthurus chirurgus* (11 individuals in 1 videos). Those species are found with higher abundance in southern location of the Brazilian coast such as Abrolhos, Bahia for parrot fish and surgeon fish, or even higher latitude such as Arvoredo Island, Santa Catarina and Arraial do Cabo, Rio de Janeiro for sea chubs^134^.

The black triggerfish is mostly distributed on the northeast side of the Island. This windward pattern of distribution is facilitated by its high swimming abilities^96^. The windward side of FNA also concentrates the more complex sediment, containing reef algal-vermetid barriers along rocky shorelines. *M. niger* directly benefits from this sediment complexity as it lays its eggs in the sand, feeds on epilithic algal matrix (EAM) and sleeps in rocky reef^137^. Adult, *M. niger* is strictly reef-associated as it sleeps in the same hole every night, which attests to a high level of site-fidelity^138^. If the bulk of the *M. niger* occurred in the selected area for biomass estimation, we also observed *M. niger* on the leeward side close to the shore where large rocks occur. In this case, *M. niger* were juvenile associated with other reef fish (e.g. surgeons fish, sergeants). Although abundant, easily catchable, edible and appetent, the black triggerfish is not a commercial species and is only consumed occasionally.

### Revisiting the Island mass effect (IME)

**Figure 10:**
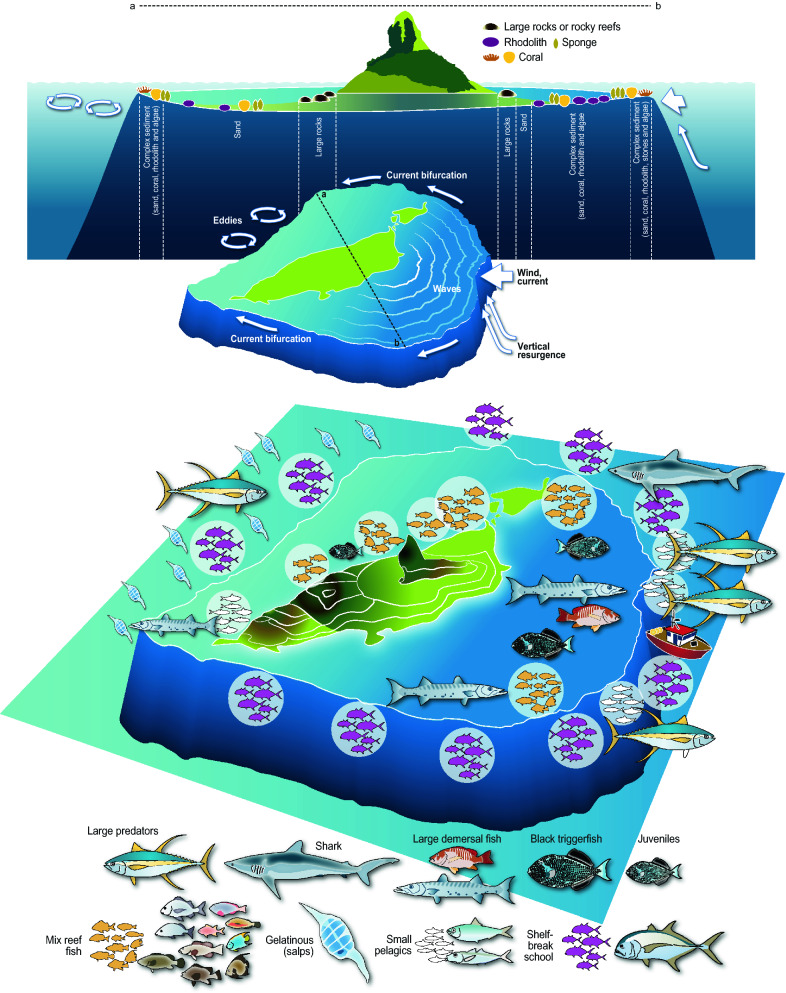
Synthetic representation of the island mass effect as illustrated by the case of Fernando de Noronha.

The IME describes well the higher primary and secondary productivity on the leeside due to turbulent mixing and advection created by eddies on the wake of islands^139–141^. However, the IME on tertiary productivity remains quite unknown or is described as isotrope around the island^27^. Our data allows us to better describe the IME and propose a new conceptual figure (Fig. 10) with an asymmetrical response of the fish distribution.

Horizontal fish distribution patterns were very different regarding the position of the archipelago face to the main wind/current flows (windward vs leeward). Overall, fish were much more abundant on the windward side. This is true for most assemblages, especially for pelagic fish and triggerfishes. According to the IME, an overall increase in island-related production is expected. This effect is expected to be more marked on the leeward side due to hydrodynamic retention processes^27,29,34,142,143^. The no-fish acoustic biomass, mostly composed of zooplankton (including gelatinous) was indeed higher on the leeward, in particular off the shelf where dense layers of gelatinous were observed. Lessa et al.^74^ observed a more important concentration of larvae on the west side (leeward) of FNA. Thus, leeward side primary productivity enhancement^34^ (see also vertical profiles of chlorophyll concentration performed during the FAROFA surveys showing higher concentration in the leeward side; 10.17882/70647) may participate in fish larvae recruitment where the leeside fulfils the conditions of Bakun triade: (i) nutrient enrichment, (ii) concentration of larval food distributions, and (iii) local retention of eggs and larvae ^144,145^.

If the IME is well described in terms of turbulent processes and further primary productivity^30^, there is a lack of fine scale information on the consequences in terms of fish distribution. We reveal that most pelagic fish are concentrated windward facing the main flow, where the productivity is expected to be lower. This study does not allow concluding about the mechanisms explaining this feature, but we can propose at least three hypotheses. First, the current flow reaching the island topography likely concentrates the flux of particles^143^ including zooplankton, favouring the feeding behaviour of medium size planktivorous pelagic fish (e.g. *C. sufflamen*) or intermediate predators that feed on small fish, shrimps or invertebrates (e.g. *Caranx crysos*) and are targeted by top predators (e.g. large Scombridae and Carangidae, Istiophoridae and sharks). The aggregation of planktivorous fish on the windward side of island has been observed by Hamner et al.^146^ that described a “wall of mouth” composed by planktivorous fish picking up zooplankton before it reaches the reef^146,147^. Second, the water is much more turbid leeward than windward where visual predators concentrate. Third, a behavioural pattern consisting in facing the current (reotrope) to keep associated to the island and avoid advection may also play a role.

Interestingly, most demersal assemblages were also more abundant on the windward side. Structural habitat complexity is known to be an important factor for fish richness and abundance^24,148,149^ and higher acoustic biomass was associated with the more complex sediment SaStCoRhAl, a mosaic of different substrates. This may be a consequence of turbulent processes. Indeed, on the windward side, the strong oceanic flow flush soft sediments, favouring the development of complex habitats that concentrate fish^148^. On the other hand, soft sediments deposit leeward, forming sandy habitats that are less populated^148^. Wind and current exposure influence the underwater landscape with the windward side, characterized by extensive reef barriers along rocky shorelines, and the leeward side, mainly composed of descending slopes along a rocky shoreline with large scattered boulders and small reefs scattered on sandy habitats^150^. This difference in sediment between the windward leeward due to the wind and wave exposure is a common feature observed elsewhere. e.g., in the Madeira Archipelago^139^.

IME effect is thus not just an enhancement of primary productivity though physical process in the leeward side of islands but it also drives the fish distribution by shaping the habitats. Wind and current cause erosion and a transformation of the shoreline and sediment distribution which also impacts the distribution of fish. This results in an anisotropic distribution of fish with schools of small pelagic and associated predators flourishing at the shelf-break on the windward side taking advantage of the vertical mixing, the current and the clear water.

### Depth strata

Fish acoustic biomass and assemblage composition varied according to the depth strata. The highest overall fish acoustic biomass was observed on the mid mesophotic zone (60–80 m) that corresponds to the shelf-break. By providing a cross-shore along-depth acoustic biomass estimations of demersal and pelagic fish, we confirm the importance of the bathymetry^151^ and quantify and rank the fish distribution among depth strata. Specifically, the shelf-break was a hot spot for five assemblages (Fig. 6). Indeed, at the shelf-break, we observed the steady presence of shelf-break schools as well as small pelagics and predators. It is in this area, in particular at the windward, that SSF targeting both demersal and pelagic species concentrate^152^. With our results, it is difficult to determine whether the depth and associated light conditions (euphotic vs mesophotic) or the structural characteristic of the vertical zones are the main drivers of diversity and abundance. Indeed, in our case the mid mesophotic zone matches the shelf-break. This zone has specific characteristics. It presents a steep slope associated to high structural complexity^18^. In addition, by being at the interface of neritic and oceanic domains it concentrates organisms from both domains^153^. Mesophotic reefs associated to the shelf-break are thus hot spots for marine life. However, our result do not confirm that mesophotic reefs per se (independently of the shelf-break) concentrate more life than euphotic ones. Despite their importance, shelf edge reefs are seldom included in marine protected area network, in particular in Brazil^23^. Shelf-breaks should thus be considered as important area for biodiversity conservation^24^.

### Insights for marine spatial planning

MSP is a natural extension of practices including integrated coastal management and multi-use MPA management^36^. MPAs are regarded by many marine scientists as a major management tool needed to tackle fisheries collapse and regular loss of marine biodiversity^154–156^. Data and analysis should be central to decision-making. The data we provide here have the advantage of being comprehensive. We show that fish acoustic biomass was significantly higher inside than outside the no-take zone (Supplementary Fig. S6 online) indicating a potential effect of the MPA. Such positive effect was witnessed by Ilarri et al.^115^ on shallow-reef fish communities. Here, by providing a comprehensive covering of the entire FNA we provide much more robust estimates. If most assemblages had higher acoustic biomass inside than outside the no-take MPA, it is difficult to fully unravel between protection effects and habitat characteristics. Indeed the no-take zone encompasses the entire windward shelf that is characterised by higher bottom habitat complexity while the no-take MPA area encompasses the area close to the port that is susceptible to enhance the productivity through the eutrophication and the presence of rocks and structures. In all cases, with the objective to protect fish biomass and assemblages, the no-take zone seems overall well designed. It covers most of the shelf, protecting reef fish that exhibit high densities in near shore shallow waters areas that classically suffer great anthropic pressure linked to multiple use activities including artisanal and recreational fishing. However, the no-take zone stops at the 50 m isobaths and thus leaves the shelf-break unprotected. As an important zone for biodiversity, foraging and spawning, protecting the shelf-break could favour species reliant on shelf-break mesophotic reefs. However, since most SSF operate at the shelf-break it is important to let a sufficient portion of the shelf-break open to fisheries, in particular for those targeting pelagic species that temporally use FNA as a shelter during their long journey.

## Conclusion

By combining multifrequency acoustic data and video, we provide the first comprehensive description of demersal and pelagic fish distribution of a tropical ecosystem. We also provide the first biomass estimation of the black triggerfish *Melichthys niger*, a key tropical player. More generally, we pictured the distribution of a variety of fish assemblages and related their spatial patterns to biotic and abiotic environmental features. Comparing the effects of euphotic and mesophotic reefs we show that more than the depth, the most important feature is the topography with the shelf-break as the most important hotspot. Beyond, this approach allowed us to revisit the IME. We completed the IME portrait and revealed that it is an asymmetric process regarding fish distribution. Indeed, while primary productivity is mostly enhanced in the leeward, higher trophic levels concentrate on the windward side. We also tested for the impact of the no-take MPA of Fernando de Noronha Archipelago on the distribution and acoustic biomass of demersal and pelagic fishes. This MPA protects the most complex habitats that shelter the highest fish diversity and biomass. Still, an important hot spot, the shelf-break, currently unprotected could be partly included given that sufficient space is left for fishing activities, in particular for pelagics. Maintaining pelagic small-scale fisheries in FNA is indeed socially and economically important. More generally, describing fish distribution and associated environmental features is the first step toward understanding how fish communities are spatially structured and is a necessary step to conduct MSP and operate relevant protection policies.

## Supplementary Information

Supplementary Information.

## Data Availability

Video annotations, CTD data and a degraded resolution of acoustic data are available online on SEANOE Sea scientific open data publication site (10.17882/76019; 10.17882/71024, 10.17882/70647).

## References

[1] Bowen BW, Rocha LA, Toonen RJ, Karl SA (2013). The origins of tropical marine biodiversity. Trends Ecol. Evol..

[2] Lam VW (2020). Climate change, tropical fisheries and prospects for sustainable development. Nat. Rev. Earth Environ..

[3] Halpern BS (2019). Recent pace of change in human impact on the world’s ocean. Sci. Rep..

[4] Capitani L, de Araujo JN, Vieira EA, Angelini R, Longo GO (2021). Ocean warming will reduce standing biomass in a tropical western atlantic reef ecosystem. Ecosystems.

[5] Lima LS (2021). Potential changes in the connectivity of marine protected areas driven by extreme ocean warming. Sci. Rep..

[6] Sale PF (2014). Transforming management of tropical coastal seas to cope with challenges of the 21st century. Mar. Pollut. Bull..

[7] Dunstan PK (2018). How can climate predictions improve sustainability of coastal fisheries in Pacific Small-Island Developing States?. Mar. Policy.

[8] Martins IM, Gasalla MA (2018). Perceptions of climate and ocean change impacting the resources and livelihood of small-scale fishers in the South Brazil Bight. Clim. Change.

[9] Moura RL (2013). Spatial patterns of benthic megahabitats and conservation planning in the Abrolhos Bank. Cont. Shelf Res..

[10] Lesser MP, Slattery M, Leichter JJ (2009). Ecology of mesophotic coral reefs. J. Exp. Mar. Biol. Ecol..

[11] Bryan DR, Kilfoyle K, Gilmore RG, Spieler RE (2013). Characterization of the mesophotic reef fish community in south Florida, USA. J. Appl. Ichthyol..

[12] Fukunaga A, Kosaki RK, Wagner D, Kane C (2016). Structure of Mesophotic Reef Fish Assemblages in the Northwestern Hawaiian Islands. PLoS One.

[13] Kahng S, Copus JM, Wagner D, Rossi S, Bramanti L, Gori A, Orejas C (2016). Mesophotic coral ecosystems. Marine Animal Forests.

[14] Rocha LA (2018). Mesophotic coral ecosystems are threatened and ecologically distinct from shallow water reefs. Science.

[15] Bongaerts P (2017). Deep reefs are not universal refuges: Reseeding potential varies among coral species. Sci. Adv..

[16] Rosa MR (2016). Mesophotic reef fish assemblages of the remote St. Peter and St. Paul’s Archipelago, Mid-Atlantic Ridge, Brazil. Coral Reefs.

[17] Medeiros AP (2021). Deep reefs are not refugium for shallow-water fish communities in the southwestern Atlantic. Ecol. Evol..

[18] Reid DG (2001). SEFOS—Shelf edge fisheries and oceanography studies: An overview. Fish. Res..

[19] Heyman WD, Kjerfve B (2008). Characterization of transient multi-species reef fish spawning aggregations at Gladden Spit, Belize. Bull. Mar. Sci..

[20] Paxton AB (2021). Four decades of reef observations illuminate deep-water grouper hotspots. Fish Fish..

[21] Frédou T, Ferreira BP (2005). Bathymetric trends of northeastern Brazilian snappers (Pisces, Lutjanidae): Implications for the reef fishery dynamic. Braz. Arch. Biol. Technol..

[22] Longhurst AR, Pauly D (2007). Ecologia dos oceanos tropicais.

[23] Olavo G, Costa PA, Martins AS, Ferreira BP (2011). Shelf-edge reefs as priority areas for conservation of reef fish diversity in the tropical Atlantic. Aquat. Conserv. Mar. Freshw. Ecosyst..

[24] Eduardo LN (2018). Identifying key habitat and spatial patterns of fish biodiversity in the tropical Brazilian continental shelf. Cont. Shelf Res..

[25] Silva MB, Rosa RS, Menezes R, Francini-Filho RB (2021). Changes in reef fish assemblages in a cross-shelf euphotic-mesophotic gradient in tropical SW Atlantic. Estuar. Coast. Shelf Sci..

[26] Doty MS, Oguri M (1956). The island mass effect. ICES J. Mar. Sci..

[27] Gove JM (2016). Near-island biological hotspots in barren ocean basins. Nat. Commun..

[28] Letessier TB (2019). Remote reefs and seamounts are the last refuges for marine predators across the Indo-Pacific. PLoS Biol..

[29] Heywood KJ, Barton ED, Simpson JH (1990). The effects of flow disturbance by an oceanic island. J. Mar. Res..

[30] Signorini SR, McClain CR, Dandonneau Y (1999). Mixing and phytoplankton bloom in the wake of the Marquesas Islands. Geophys. Res. Lett..

[31] Henry, G. W. & Lyle, J. M. National recreational and indigenous fishing survey (2003).

[32] Coutis PF, Middleton JH (1999). Flow-topography interaction in the vicinity of an isolated, deep ocean island. Deep Sea Res. Part Oceanogr. Res. Pap..

[33] Cardoso C, Caldeira RMA, Relvas P, Stegner A (2020). Islands as eddy transformation and generation hotspots: Cabo Verde case study. Prog. Oceanogr..

[34] Tchamabi CC, Araujo M, Silva M, Bourlès B (2017). A study of the Brazilian Fernando de Noronha island and Rocas atoll wakes in the tropical Atlantic. Ocean Model.

[35] Motta FS (2021). Effects of marine protected areas under different management regimes in a hot spot of biodiversity and cumulative impacts from SW Atlantic. Reg. Stud. Mar. Sci..

[36] Agardy T, di Sciara GN, Christie P (2011). Mind the gap: Addressing the shortcomings of marine protected areas through large scale marine spatial planning. Mar. Policy.

[37] Shucksmith RJ, Kelly C (2014). Data collection and mapping—Principles, processes and application in marine spatial planning. Mar. Policy.

[38] Queffelec B (2021). Marine spatial planning and the risk of ocean grabbing in the tropical Atlantic. ICES J. Mar. Sci..

[39] Rubio-Cisneros NT (2019). Poor fisheries data, many fishers, and increasing tourism development: Interdisciplinary views on past and current small-scale fisheries exploitation on Holbox Island. Mar. Policy.

[40] Samhouri JF, Haupt AJ, Levin PS, Link JS, Shuford R (2014). Lessons learned from developing integrated ecosystem assessments to inform marine ecosystem-based management in the USA. ICES J. Mar. Sci..

[41] Long RD, Charles A, Stephenson RL (2015). Key principles of marine ecosystem-based management. Mar. Policy.

[42] Hewitt JE, Anderson MJ, Thrush SF (2005). Assessing and monitoring ecological community health in marine systems. Ecol. Appl..

[43] Caselle JE, Rassweiler A, Hamilton SL, Warner RR (2015). Recovery trajectories of kelp forest animals are rapid yet spatially variable across a network of temperate marine protected areas. Sci. Rep..

[44] Díaz-Pérez L (2016). Coral Reef Health Indices versus the Biological, Ecological and Functional Diversity of Fish and Coral Assemblages in the Caribbean Sea. PLoS One.

[45] Topor ZM, Rasher DB, Duffy JE, Brandl SJ (2019). Marine protected areas enhance coral reef functioning by promoting fish biodiversity. Conserv. Lett..

[46] Pennino MG (2016). Fishery-dependent and -independent data lead to consistent estimations of essential habitats. ICES J. Mar. Sci..

[47] Hilborn R, Walters CJ (2013). Quantitative Fisheries Stock Assessment: Choice, Dynamics and Uncertainty.

[48] Bohnsack, J. A. & Bannerot, S. P. A stationary visual census technique for quantitatively assessing community structure of coral reef fishes (1986).

[49] Jones RS, Thompson MJ (1978). Comparison of Florida reef fish assemblages using a rapid visual technique. Bull. Mar. Sci..

[50] Kimmel JJ (1985). A new species-time method for visual assessment of fishes and its comparison with established methods. Environ. Biol. Fishes.

[51] Michalopoulos C, Auster PJ, Malatesta RJ (1992). A comparison of transect and species-time counts for assessing faunal abundance from video surveys. Mar. Technol. Soc. J..

[52] Gray JS, Ugland KI, Lambshead J (2004). Species accumulation and species area curves: A comment on Scheiner (2003). Glob. Ecol. Biogeogr..

[53] Mallet D, Pelletier D (2014). Underwater video techniques for observing coastal marine biodiversity: A review of sixty years of publications (1952–2012). Fish. Res..

[54] Langlois TJ (2010). Cost-efficient sampling of fish assemblages: Comparison of baited video stations and diver video transects. Aquat. Biol..

[55] Logan JM, Young MA, Harvey ES, Schimel ACG, Ierodiaconou D (2017). Combining underwater video methods improves effectiveness of demersal fish assemblage surveys across habitats. Mar. Ecol. Prog. Ser..

[56] Koslow JA (2009). The role of acoustics in ecosystem-based fishery management. ICES J. Mar. Sci..

[57] Bertrand A (2014). Broad impacts of fine-scale dynamics on seascape structure from zooplankton to seabirds. Nat. Commun..

[58] Benoit-Bird KJ, Lawson GL (2016). Ecological insights from pelagic habitats acquired using active acoustic techniques. Annu. Rev. Mar. Sci..

[59] Sutton TT (2013). Vertical ecology of the pelagic ocean: Classical patterns and new perspectives. J. Fish Biol..

[60] McClatchie S, Thorne RE, Grimes P, Hanchet S (2000). Ground truth and target identification for fisheries acoustics. Fish. Res..

[61] Cappo M, Speare P, De’ath G (2004). Comparison of baited remote underwater video stations (BRUVS) and prawn (shrimp) trawls for assessments of fish biodiversity in inter-reefal areas of the Great Barrier Reef Marine Park. J. Exp. Mar. Biol. Ecol..

[62] Harvey ES, Cappo M, Butler JJ, Hall N, Kendrick GA (2007). Bait attraction affects the performance of remote underwater video stations in assessment of demersal fish community structure. Mar. Ecol. Prog. Ser..

[63] Fitzpatrick BM, Harvey ES, Heyward AJ, Twiggs EJ, Colquhoun J (2012). Habitat specialization in tropical continental shelf demersal fish assemblages. PLoS One.

[64] Rooper CN, Hoff GR, De Robertis A (2010). Assessing habitat utilization and rockfish (*Sebastes* spp.) biomass on an isolated rocky ridge using acoustics and stereo image analysis. Can. J. Fish. Aquat. Sci..

[65] Jones, D. *et al.* Evaluation of rockfish abundance in untrawlable habitat: Combining acoustic and complementary sampling tools (2012).

[66] O’Driscoll RL (2012). Species identification in seamount fish aggregations using moored underwater video. ICES J. Mar. Sci..

[67] Fernandes PG, Copland P, Garcia R, Nicosevici T, Scoulding B (2016). Additional evidence for fisheries acoustics: Small cameras and angling gear provide tilt angle distributions and other relevant data for mackerel surveys. ICES J. Mar. Sci..

[68] Gastauer S, Scoulding B, Parsons M (2017). An unsupervised acoustic description of fish schools and the seabed in three fishing regions within the Northern Demersal Scalefish Fishery (NDSF, Western Australia). Acoust. Aust..

[69] Blanluet A (2019). Characterization of sound scattering layers in the Bay of Biscay using broadband acoustics, nets and video. PLoS One.

[70] Campanella F, Taylor JC (2016). Investigating acoustic diversity of fish aggregations in coral reef ecosystems from multifrequency fishery sonar surveys. Fish. Res..

[71] Domokos R (2021). On the development of acoustic descriptors for semi-demersal fish identification to support monitoring stocks. ICES J. Mar. Sci..

[72] Villalobos H (2021). A practical approach to monitoring marine protected areas: An application to El Bajo Espíritu Santo Seamount near La Paz, Mexico. Oceanography.

[73] Hazin FH, Zagaglia JR, Broadhurst MK, Travassos PEP, Bezerra TRQ (1998). Review of a small-scale pelagic longline fishery off northeastern Brazil. Mar. Fish. Rev..

[74] Lessa RP (1999). Distribution and abundance of ichthyoneuston at seamounts and islands off north-eastern Brazil. Arch. Fish. Mar. Res..

[75] Dominguez PS, Zeineddine GC, Rotundo MM, Barrella W, Ramires M (2014). A pesca artesanal no arquipélago de Fernando de Noronha (PE). Bol. Inst. Pesca.

[76] Lopes PFM, Mendes L, Fonseca V, Villasante S (2017). Tourism as a driver of conflicts and changes in fisheries value chains in Marine Protected Areas. J. Environ. Manag..

[77] Outeiro L, Rodrigues JG, Damásio LMA, Lopes PFM (2019). Is it just about the money? A spatial-economic approach to assess ecosystem service tradeoffs in a marine protected area in Brazil. Ecosyst. Serv..

[78] Garla RC, Chapman DD, Wetherbee BM, Shivji M (2006). Movement patterns of young Caribbean reef sharks, Carcharhinus perezi, at Fernando de Noronha Archipelago, Brazil: The potential of marine protected areas for conservation of a nursery ground. Mar. Biol..

[79] Bertrand A (2017). FAROFA 1 cruise. RV TUBARAO Tigre..

[80] Bertrand A (2018). FAROFA 2 cruise. RV TUBARAO Tigre..

[81] Bertrand A (2019). FAROFA 3 cruise. RV TUBARAO Tigre..

[82] Bertrand, A. *et al.* Acoustic data from FAROFA surveys, 2017-09-15 to 2019-04-22. 10.17882/71024 (2020).

[83] Salvetat, J. *et al.* Underwater video observations from FAROFA surveys, 2017-09-15 to 2019-04-22. 10.17882/76019 (2020).

[84] Pawlowicz, R. M_Map: A mapping package for MATLAB, version 1.4 m (computer software) (2020).

[85] Péter, A. *Solomon Coder: The Concept of Behavioral Elements, Categories and the Representation of Data in Solomon Coder* (2019).

[86] Priede IG, Bagley PM, Smith A, Creasey S, Merrett NR (1994). Scavenging deep demersal fishes of the Porcupine Seabight, north-east Atlantic: Observations by baited camera, trap and trawl. J. Mar. Biol. Assoc. U. K..

[87] McQuinn IH (2005). Description of the ICES HAC standard data exchange format, version 1.60.

[88] Trenkel VM (2009). Overview of recent progress in fisheries acoustics made by Ifremer with examples from the Bay of Biscay. Aquat. Living Resour..

[89] Perrot Y (2018). Matecho: An open-source tool for processing fisheries acoustics data. Acoust. Aust..

[90] Salvetat J (2020). In situ target strength measurement of the black triggerfish *Melichthys*
*niger* and the ocean triggerfish *Canthidermis*
*sufflamen*. Mar. Freshw. Res..

[91] Lavery AC (2007). Determining dominant scatterers of sound in mixed zooplankton populations. J. Acoust. Soc. Am..

[92] MacLennan DN, Fernandes PG, Dalen J (2002). A consistent approach to definitions and symbols in fisheries acoustics. ICES J. Mar. Sci..

[93] Barros MJG (2020). Analises da Ictiofauna marinha e habitats associados atraves de videos subaquatica.

[94] Sazima C, Bonaldo RM, Krajewski JP, Sazima I (2005). The Noronha wrasse: A jack-of-all-trades follower. Aqua J. Ichthyol. Aquat. Biol..

[95] Soto JMR (2001). Peixes do arquipélago Fernando de Noronha. Mare Magnum.

[96] Krajewski JP, Floeter SR (2011). Reef fish community structure of the Fernando de Noronha Archipelago (Equatorial Western Atlantic): The influence of exposure and benthic composition. Environ. Biol. Fishes.

[97] Sazima I, Sazima C, da Silva-Jr JM (2006). Fishes associated with spinner dolphins at Fernando de Noronha Archipelago, tropical Western Atlantic: An update and overview. Neotropical Ichthyol..

[98] Petitgas P (1993). Use of a disjunctive kriging to model areas of high pelagic fish density in acoustic fisheries surveys. Aquat. Living Resour..

[99] Chiles J-P, Delfiner P (2009). Geostatistics: Modeling Spatial Uncertainty.

[100] Bez N, Braham C-B (2014). Indicator variables for a robust estimation of an acoustic index of abundance. Can. J. Fish. Aquat. Sci..

[101] Switzer, P. Min/max autocorrelation factors for multivariate spatial imagery. *Comput. Sci. Stat.* (1985).

[102] Bez, N. Global estimation based on indicators factorization (2021).

[103] Assunção RV, Silva AC, Martins J, Montes MF (2016). Spatial-temporal variability of the thermohaline properties in the coastal region of Fernando de Noronha Archipelago, Brazil. J. Coast. Res..

[104] da Silva AC (2021). Surface circulation and vertical structure of upper ocean variability around Fernando de Noronha archipelago and Rocas atoll during spring 2015 and fall 2017. Front. Mar. Sci..

[105] Breiman L, Friedman J, Olshen R, Stone C (1984). Classification and regression trees. Wadsworth Int. Group.

[106] Therneau, T., Atkinson, B., Ripley, B. & Ripley, M. B. Package ‘rpart’. Available Online Cran Ma Ic Ac Ukwebpackagesrpartrpart Pdf Accessed 20 April 2016 (2015).

[107] Kuhnert PM, Duffy LM, Young JW, Olson RJ (2012). Predicting fish diet composition using a bagged classification tree approach: A case study using yellowfin tuna (*Thunnus albacares*). Mar. Biol..

[108] Breiman L (1996). Bagging predictors. Mach. Learn..

[109] Kuhnert PM, Henderson A-K, Bartley R, Herr A (2010). Incorporating uncertainty in gully erosion calculations using the random forests modelling approach. Environmetrics.

[110] Kuhnert PM, Mengersen K (2003). Reliability measures for local nodes assessment in classification trees. J. Comput. Graph. Stat..

[111] R Core Team. *R: A language and environment for statistical computing* (2020).

[112] ParisTech, M. ARMINES: RGeostats: The Geostatistical R Package (2020).

[113] Kahle DJ, Wickham H (2013). ggmap: Spatial visualization with ggplot2. R J.

[114] Pimentel, C. R. *et al.* Mesophotic ecosystems at Fernando de Noronha Archipelago, Brazil (South-western Atlantic), reveal unique ichthyofauna and need for conservation. *Neotropical Ichthyol.* **18** (2020).

[115] Ilarri MI, Souza AT, Rosa RS (2017). Community structure of reef fishes in shallow waters of the Fernando de Noronha archipelago: Effects of different levels of environmental protection. Mar. Freshw. Res..

[116] Schmid, K. *et al.* First fish fauna assessment in the Fernando de Noronha Archipelago with BRUVS: Species catalog with underwater imagery. *Biota Neotropica* **20** (2020).

[117] de Araújo ME (2020). Diversity patterns of reef fish along the Brazilian tropical coast. Mar. Environ. Res..

[118] Krajewski JP, Floeter SR, Jones GP, Leite FP (2011). Patterns of variation in behaviour within and among reef fish species on an isolated tropical island: Influence of exposure and substratum. J. Mar. Biol. Assoc. U. K..

[119] Mendes TC, Quimbayo JP, Bouth HF, Silva LP, Ferreira CE (2019). The omnivorous triggerfish *Melichthys*
*niger* is a functional herbivore on an isolated Atlantic oceanic island. J. Fish Biol..

[120] Petitgas P, Levenez JJ (1996). Spatial organization of pelagic fish: Echogram structure, spatio-temporal condition, and biomass in Senegalese waters. ICES J. Mar. Sci..

[121] Burgos JM, Horne JK (2008). Characterization and classification of acoustically detected fish spatial distributions. ICES J. Mar. Sci..

[122] Russ GR (2003). Grazer biomass correlates more strongly with production than with biomass of algal turfs on a coral reef. Coral Reefs.

[123] Friedlander AM, Parrish JD (1998). Habitat characteristics affecting fish assemblages on a Hawaiian coral reef. J. Exp. Mar. Biol. Ecol..

[124] Munday PL (2002). Does habitat availability determine geographical-scale abundances of coral-dwelling fishes?. Coral Reefs.

[125] Martins K (2021). Assessing trophic interactions between pelagic predatory fish by gut content and stable isotopes analysis around Fernando de Noronha Archipelago (Brazil), Equatorial West Atlantic. J. Fish Biol..

[126] Costa B, Taylor JC, Kracker L, Battista T, Pittman S (2014). Mapping reef fish and the seascape: Using acoustics and spatial modeling to guide coastal management. PLoS One.

[127] Kavanagh KD, Olney JE (2006). Ecological correlates of population density and behavior in the circumtropical black triggerfish *Melichthys*
*niger* (Balistidae). Environ. Biol. Fishes.

[128] Lubbock R (1980). The shore fishes of Ascension Island. J. Fish Biol..

[129] Price JH, John DM (1980). Ascension Island, South Atlantic: A survey of inshore benthic macroorganisms, communities and interactions. Aquat. Bot..

[130] Robertson DR, Allen GR (1996). Zoogeography of the shorefish fauna of Clipperton Atoll. Coral Reefs.

[131] Gasparini JL, Floeter SR (2001). The shore fishes of Trindade Island, western south Atlantic. J. Nat. Hist..

[132] Lubbock R, Edwards A (1981). The fishes of Saint Paul’s rocks. J. Fish Biol..

[133] Feitoza BM, Rocha LA, Luiz-Júnior OJ, Floeter SR, Gasparini JL (2003). Reef fishes of St. Paul’s Rocks: New records and notes on biology and zoogeography. Aqua.

[134] Ferreira CEL, Floeter SR, Gasparini JL, Ferreira BP, Joyeux JC (2004). Trophic structure patterns of Brazilian reef fishes: A latitudinal comparison. J. Biogeogr..

[135] Floeter SR (2008). Atlantic reef fish biogeography and evolution. J. Biogeogr..

[136] Morais RA, Ferreira CEL, Floeter SR (2017). Spatial patterns of fish standing biomass across Brazilian reefs. J. Fish Biol..

[137] Walsh WJ (1987). Patterns of recruitment and spawning in Hawaiian reef fishes. Environ. Biol. Fishes.

[138] Walsh WJ (1984). Aspects of Nocturnal Shelter, Habitat Space, and Juvenile Recruitment in Hawaiian Coral Reef Fishes.

[139] Caldeira RMA, Groom S, Miller P, Pilgrim D, Nezlin NP (2002). Sea-surface signatures of the island mass effect phenomena around Madeira Island, Northeast Atlantic. Remote Sens. Environ..

[140] Martinez E, Maamaatuaiahutapu K (2004). Island mass effect in the Marquesas Islands: Time variation. Geophys. Res. Lett..

[141] Messié M (2020). The delayed island mass effect: How islands can remotely trigger blooms in the oligotrophic ocean. Geophys. Res. Lett..

[142] de Souza CS, da Luz JAG, Macedo S, de Montes MJF, Mafalda P (2013). Chlorophyll *a* and nutrient distribution around seamounts and islands of the tropical south-western Atlantic. Mar. Freshw. Res..

[143] Travassos P, Hazin FH, Zagaglia JR, Advíncula R, Schober J (1999). Thermohaline structure around seamounts and islands off North-Eastern Brazil. Arch. Fish. Mar. Res..

[144] Bakun, A. Ocean triads and radical interdecadal variation: Bane and boon to scientific fisheries management. in *Reinventing fisheries management* 331–358 (Springer, 1998).

[145] Agostini VN, Bakun A (2002). ‘Ocean triads’ in the Mediterranean Sea: Physical mechanisms potentially structuring reproductive habitat suitability (with example application to European anchovy, *Engraulis*
*encrasicolus*). Fish. Oceanogr..

[146] Hamner WM, Jones MS, Carleton JH, Hauri IR, Williams DM (1988). Zooplankton, planktivorous fish, and water currents on a windward reef face: Great Barrier Reef, Australia. Bull. Mar. Sci..

[147] Valenzuela J, Bellwood D, Morais R (2021). Ontogenetic habitat shifts in fusiliers (Lutjanidae): Evidence from Caesio cuning at Lizard Island, Great Barrier Reef. Coral Reefs.

[148] Curley BG, Kingsford MJ, Gillanders BM (2002). Spatial and habitat-related patterns of temperate reef fish assemblages: Implications for the design of Marine Protected Areas. Mar. Freshw. Res..

[149] Ferrari R (2018). Habitat structural complexity metrics improve predictions of fish abundance and distribution. Ecography.

[150] Maida, M. & Ferreira, B. P. Coral reefs of Brazil: An overview. in *Proceedings of the 8th International Coral Reef Symposium* Vol. 1 74 (Smithsonian Tropical Research Institute Panamá, 1997).

[151] Pittman SJ, Costa BM, Battista TA (2009). Using lidar bathymetry and boosted regression trees to predict the diversity and abundance of fish and corals. J. Coast. Res..

[152] Costa T (2019). Análise comportamental e distribuição da atividade pesqueira no Arquipelágo de Fernando de Noronha (Nordeste, BR) baseada em dados de GPS.

[153] Spalding MD (2007). Marine ecoregions of the world: A bioregionalization of coastal and shelf areas. Bioscience.

[154] Claudet J, Pelletier D, Jouvenel J-Y, Bachet F, Galzin R (2006). Assessing the effects of marine protected area (MPA) on a reef fish assemblage in a northwestern Mediterranean marine reserve: Identifying community-based indicators. Biol. Conserv..

[155] Caveen AJ, Gray TS, Stead SM, Polunin NVC (2013). MPA policy: What lies behind the science?. Mar. Policy.

[156] Hernández CM (2019). Evidence and patterns of tuna spawning inside a large no-take Marine Protected Area. Sci. Rep..

