# Supplementary material for: Comprehensive spatial distribution of tropical fish assemblages from multifrequency acoustics and video fulfils the island mass effect framework
Source: Sci Rep. 2022 May 24;12:8787. doi: 10.1038/s41598-022-12409-9 (PMC9130204; doi:10.1038/s41598-022-12409-9)
Supplement: Supplementary file 1 — Supplementary Information. [file 41598_2022_12409_MOESM1_ESM.docx]

**Supplementary material**

**to**

**Comprehensive spatial distribution of tropical fish assemblages from multifrequency acoustics and video fulfils the island mass effect framework**

Julie Salvetat^1,2,3*^, Nicolas Bez^2,3^, Jeremie Habasque^4^, Anne Lebourges-Dhaussy^4^, Cristiano Lopes^1^, Gildas Roudaut^4^, Monique Simier^2,3^, Paulo Travassos^1^, Gary Vargas^1^ and Arnaud Bertrand^2,3,1,5^

^1^Pós-Graduação em Recursos Pesqueiros e Aquicultura, Universidade Federal Rural de Pernambuco, Rua Dom Manoel de Medeiros, s/n, Dois Irmãos, Recife - PE, 52171-900, Brazil

^2^MARBEC, Univ Montpellier, CNRS, IRD, Ifremer, Sète, France

^3^Institut de Recherche pour le Développement, Sète, France

^4^IRD, LEMAR IFREMER/IRD/CNRS/UBO, Technopole Brest Iroise, Plouzané, France

^5^Laboratório de Oceanografia Física Estuarina e Costeira, Depto. Oceanografia, Universidade Federal de Pernambuco, Av. Prof. Moraes Rego, 1235 - Cidade Universitária, Recife - PE, 50670-901, Brazil

^*^Corresponding author. Email: ju.salvetat@gmail.com

**
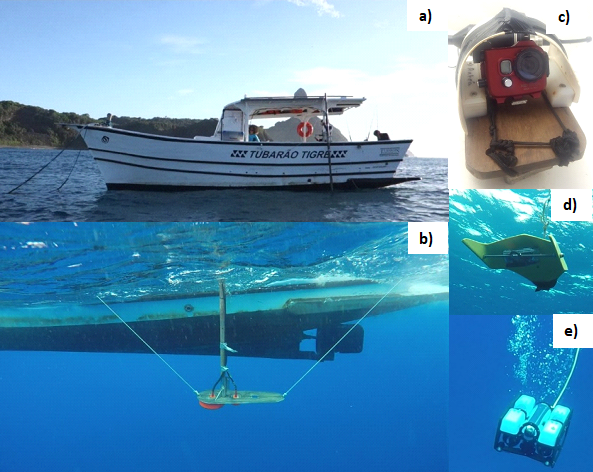
**

Supplementary Figure S1. Setting up of operations a) sport fishing boat “Tubarão tigre”; b) acoustic transducers (70 and 200 kHz) attached on a pole; c) vertical video system; d) towed video; e) ROV. Images were obtained by the authors.

Supplementary Table S1. Sediment composition name and description. Images were obtained by the authors.

| Name | Description | Example of image |
| --- | --- | --- |
| Sa | Sand only | 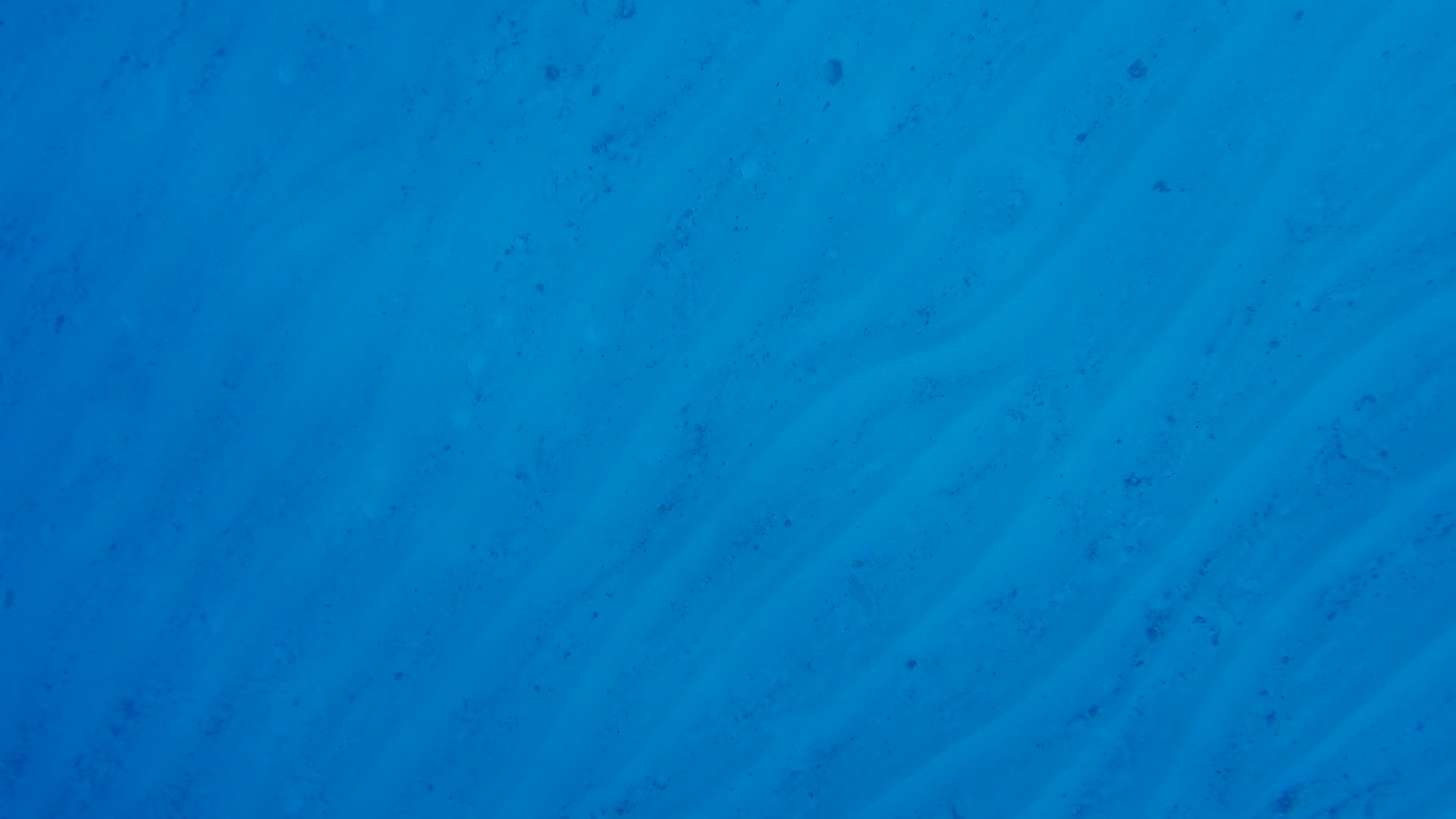 |
| LrAl | Large rocks and algae | 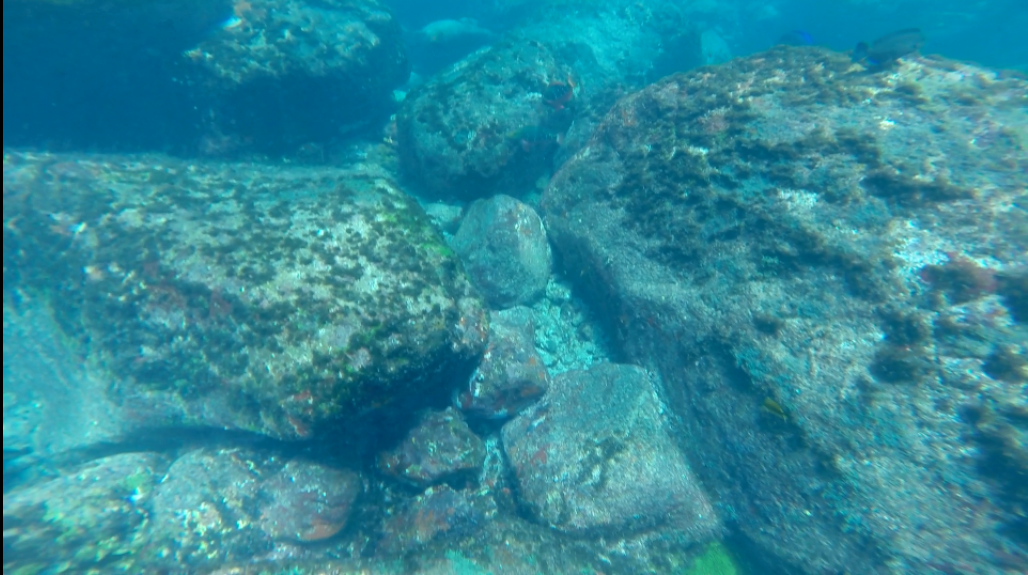 |
| SaUn | Sand and unknown elements | 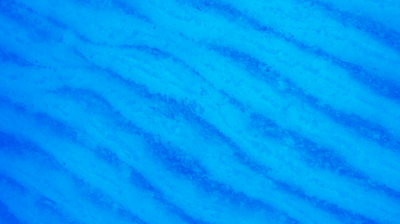 |
| SaAl | Sand and macro-algae | 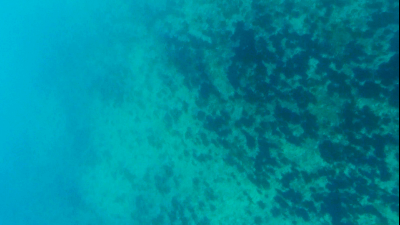 |
| SaStAl | Sand, stones and algae | 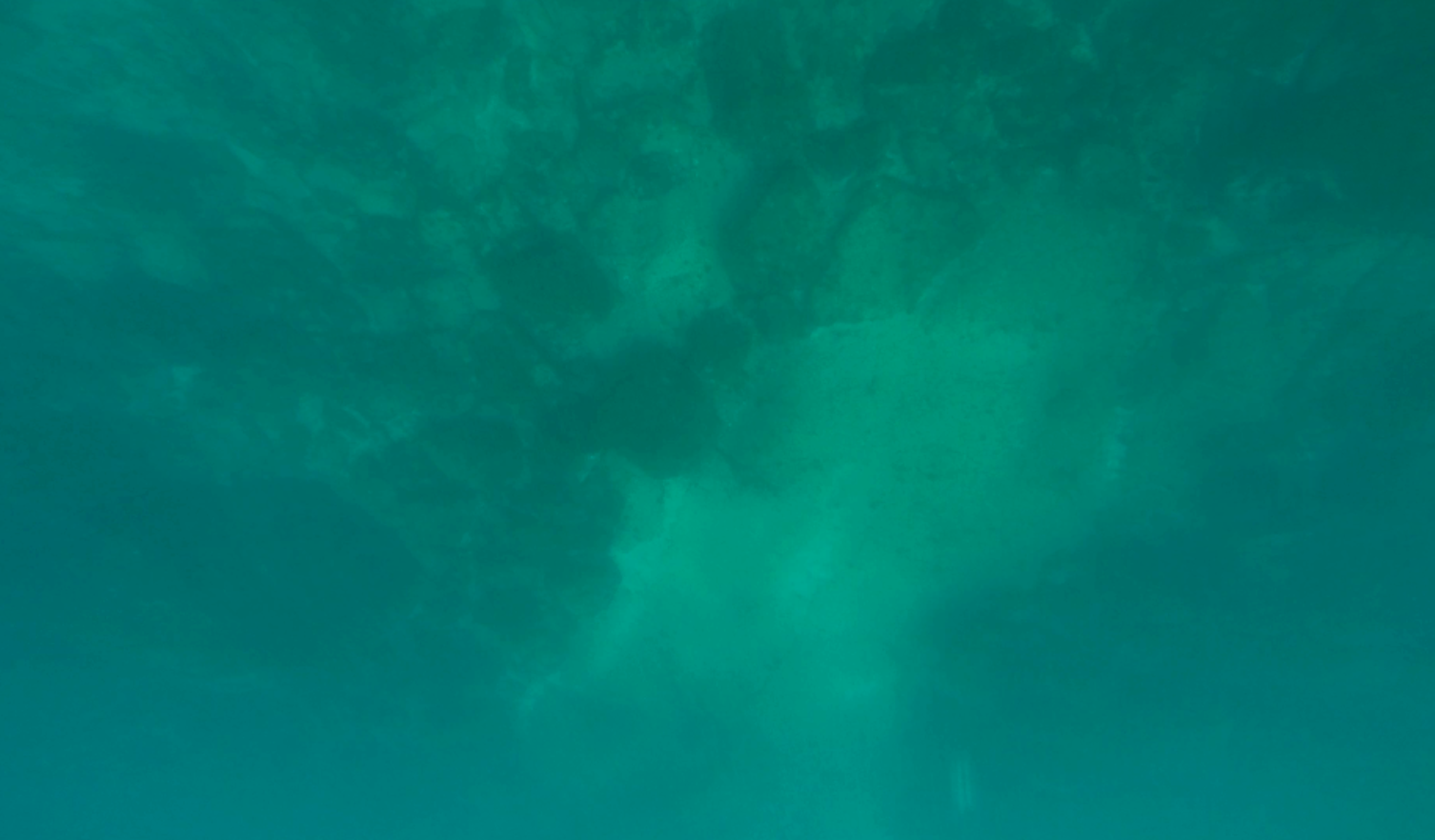 |
| SaLrAl | Sand, large rocks and algae | 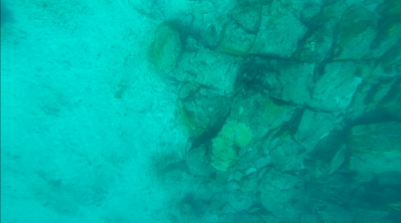 |
| SaRhAl | Sand, rhodolith and algae | 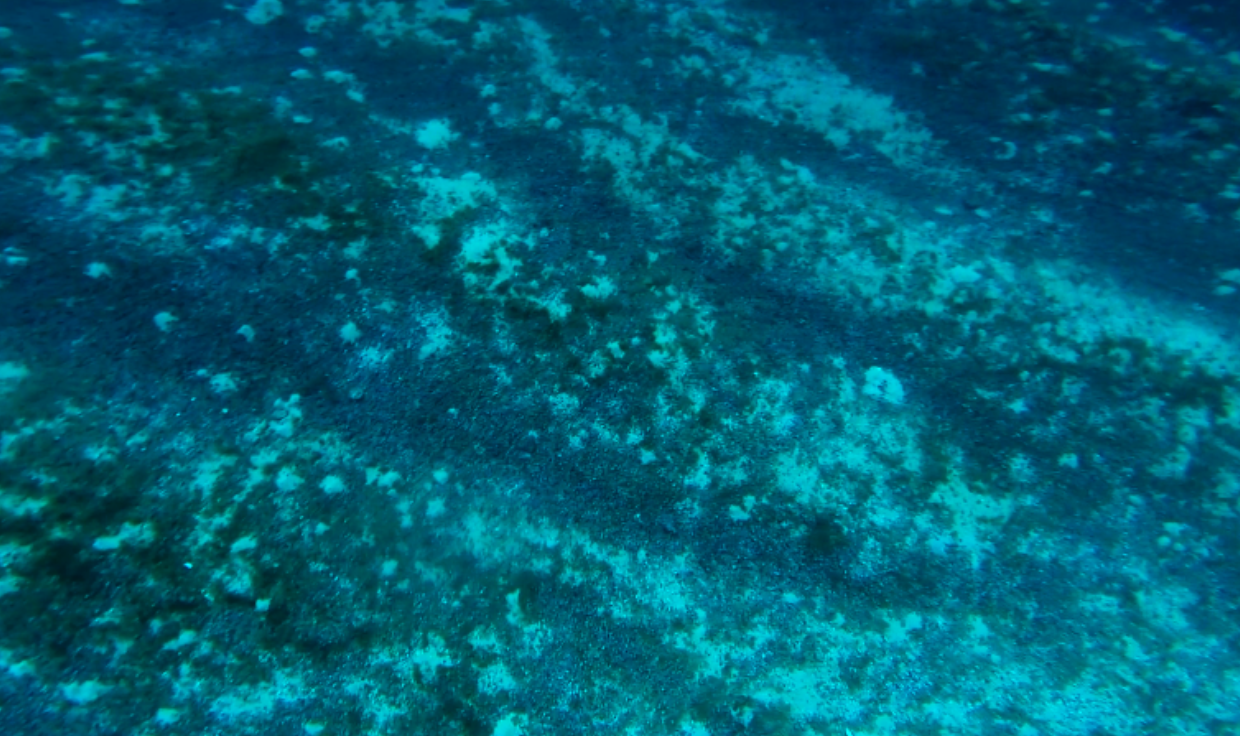 |
| SaCoRhAl | Sand, coral, rhodolith and algae | 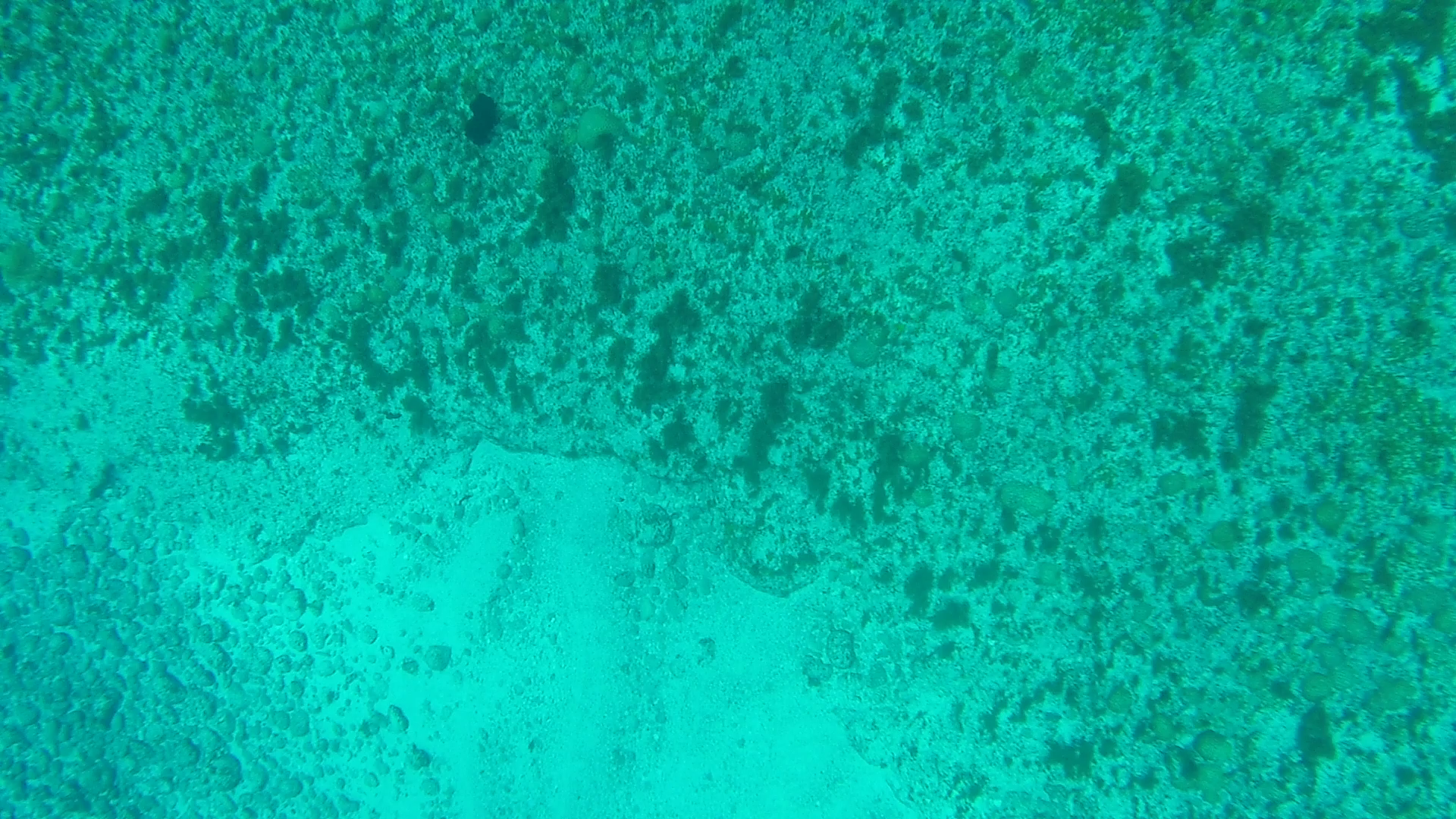 |
| SaStCoRhAl | Sand, stones, coral, rhodolith and algae | 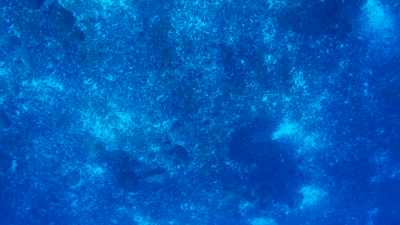 |

# Supplementary Material Methods: Acoustic data processing

Acoustic data were treated at their highest resolution i.e., 1 ping horizontally and 0.20 m vertically. To discriminate between scatters attributed to fish (fish-like) and those originated by other organisms (no-fish), e.g. gelatinous and crustaceans, we developed an approach based on thresholds on (i) volume backscattering strength Sv (Sv, in dB re 1 m^-1^; see^88^ for acoustic definitions), (ii) the bi-frequency sum of Sv, and (iii) the variance of Sv (see Supplementary Fig. S2).

A lower threshold was applied on both frequencies at -80 dB and only the data above 100 m were considered because of the limited vertical range of the 200 kHz. The first step consisted in the application of thresholds on Sv and on the variance of Sv (Supplementary Fig. S2). Thresholds on Sv were applied to select echoes strong-enough to be considered as fish. This operation was not sufficient to discriminate between fish-like and no-fish echoes. Indeed, off the shelf-break, we observed continuous homogeneous layers at ~80 m depth. Videos records showed that these layers were originated by small gelatinous, likely gas-bearing, that could not be identified. These layers had high backscattering response at both 70 and 200 kHz, preventing their discrimination from fish-like echoes using Sv thresholds only. For that reason, we added a criterion based on the variance of Sv. Indeed, the variance among elementary Sv was significantly higher in ground-thrusted fish echo-traces than in gelatinous layers, at both frequencies (F-test, p=0). Therefore, we calculated the Sv variance of the elementary cells at the centre of rectangular window of 31 x 5 cells (15 cells before and after, horizontally, and 2 cells above and below, vertically). The size of this window was defined considering the anisotropy of studied processes. Layers are by definition longer than high, while most fish shoals are higher than long in the echograms. The horizontally extended window allows minimising the variance within the layer and maximising the contrast with fish echoes. In case of dense fish school, the thresholds on Sv variance inside the moving window may eliminate the centre of a school where the variance is low. Thus, a second step (Supplementary Fig. S2) was necessary on echoes classified as no-fish at the end of the first step. It consisted of a threshold applied on the sum of Sv at both frequencies, complementing the power of discrimination between fish-like and no-fish echoes^89,90^ . This step allowed retaining the dense and homogeneous regions of fish echoes (centre of shoal or very strong isolated echoes) that could be ruled out by the variance threshold.

To estimates the thresholds, we applied a sensitivity analysis using the efficiency of the fish discrimination as criteria. The sensitivity analysis was applied over regions selected from video footages containing only gelatinous layer (3 regions) or fish shoals (3 regions). The regions with fish were treated manually to extract fish and served as reference echotrace. Sv thresholds on both frequencies were estimated by setting the variance threshold and the threshold on Sv successively for one frequency at a time for each cruise

During FAROFA 1, weather conditions were harsh and the acoustic data set was noisier and more difficult to clean so different thresholds were used. For FAROFA 1 at 70 kHz, the Sv and variance thresholds (Step 1) were set-up at -60 dB and 70 dB^2^, respectively. At 200 kHz, these thresholds were set-up at -62 dB and 90 dB^2^, respectively. Ultimately, the threshold on the sum of Sv (Step 2) was set-up at -110 kHz. For FAROFA 2 and 3 at 70 kHz, the Sv and variance thresholds (Step 1) were set-up at -65 dB and 70 dB^2^, respectively. At 200 kHz, these thresholds were set-up at -65 dB and 90 dB^2^, respectively. Ultimately, the threshold on the sum of Sv (Step 2) was set-up at -120 kHz.

To retrieve residual fish-like still present in the no-fish echograms, additional thresholds (Step 3) were used to retain values above -55 dB in Sv (70) and Sv (200). At the end of these three steps, some manual cleaning (Step 4) of fish-like echograms was necessary to eliminate some portion of gelatinous layers. After applying this process chain to the original acoustic data we obtain fish-like echograms in which we attributed a very low Sv value (-150 dB), far below the detection threshold, to the cells not containing fish-like echoes. By inverting the fish-like mask we obtained the no fish-like echograms where we attributed the same low Sv value (-150 dB) to the cells containing fish-like echoes (see Supplementary Fig.S3).


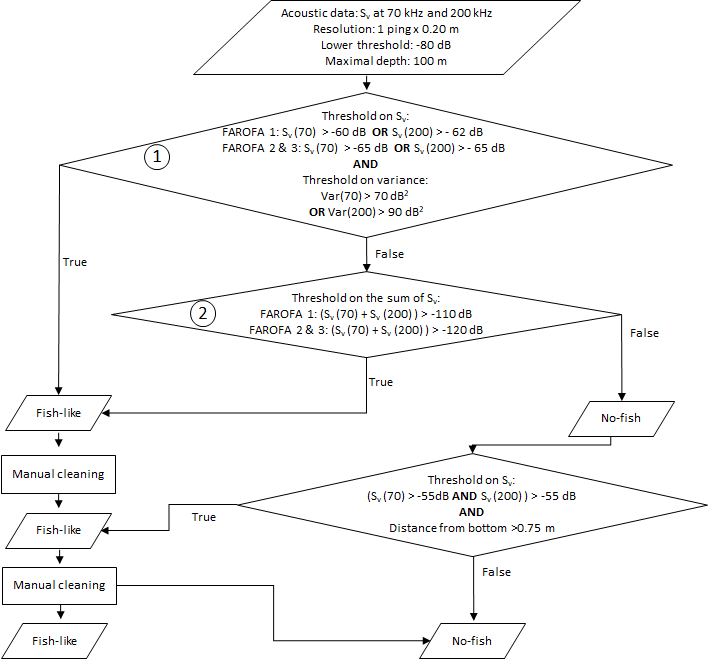


Supplementary Figure S2. Summary flowchart documenting acoustic data processing steps.


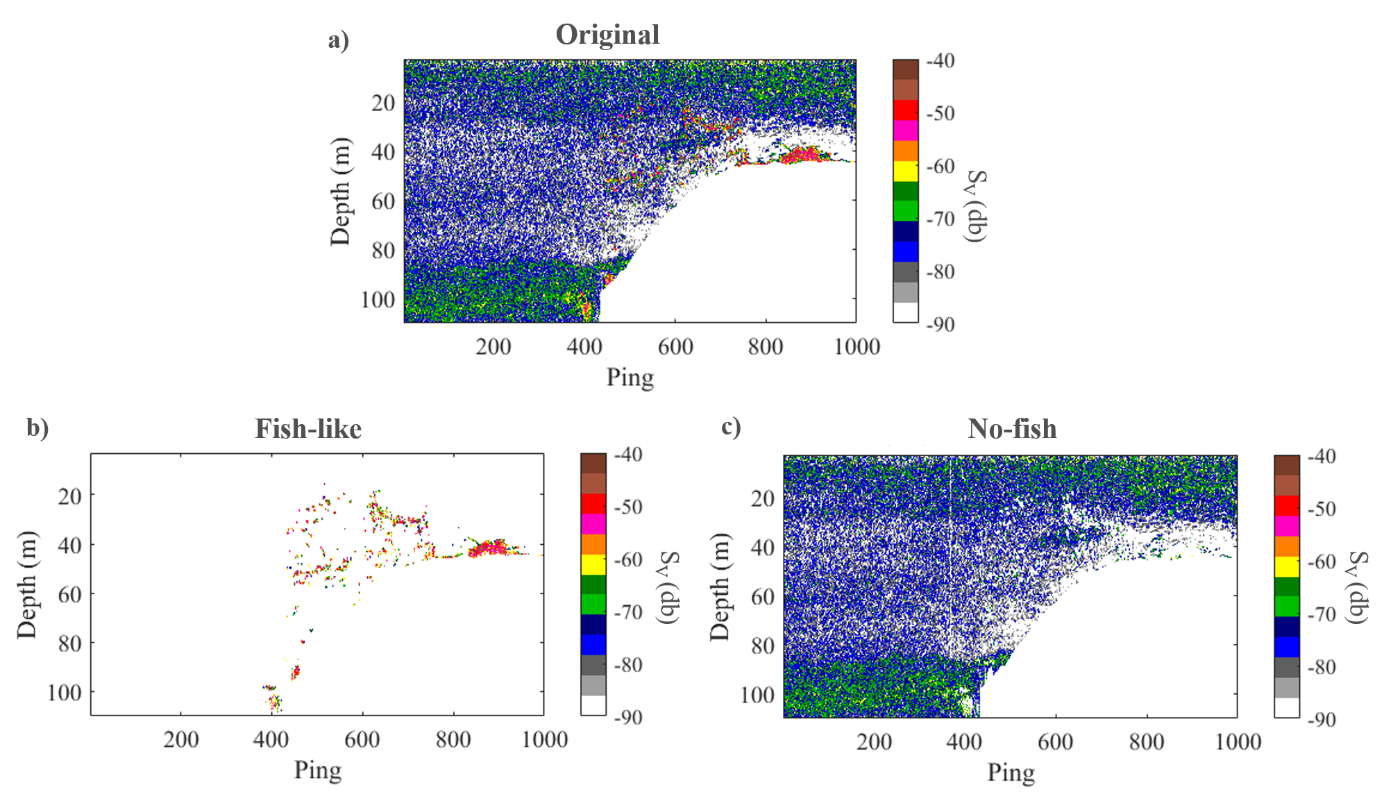


Supplementary Figure S3. Examples of an (a) original echogram and its transformation into a (b) fish-like echogram and a (c) no-fish echogram at 70 kHz.


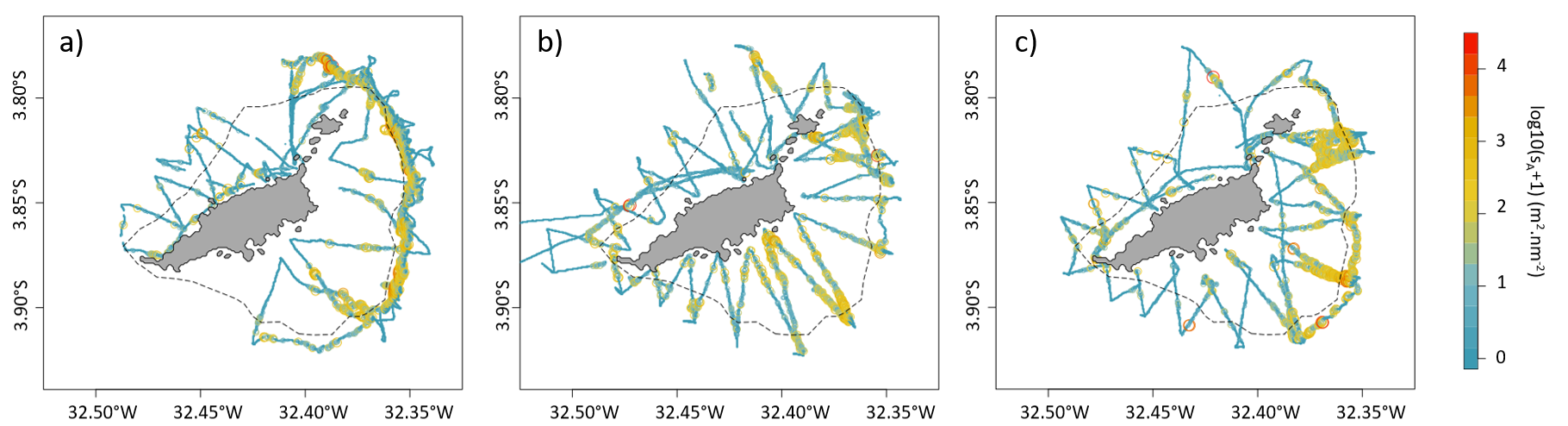


Supplementary Figure S4. Fish distribution for FAROFA (a) 1, (b) 2 and (c) 3. The dashed black line depict the 50 m isobaths. Maps were created by the authors using R (https://www.R-project.org/).


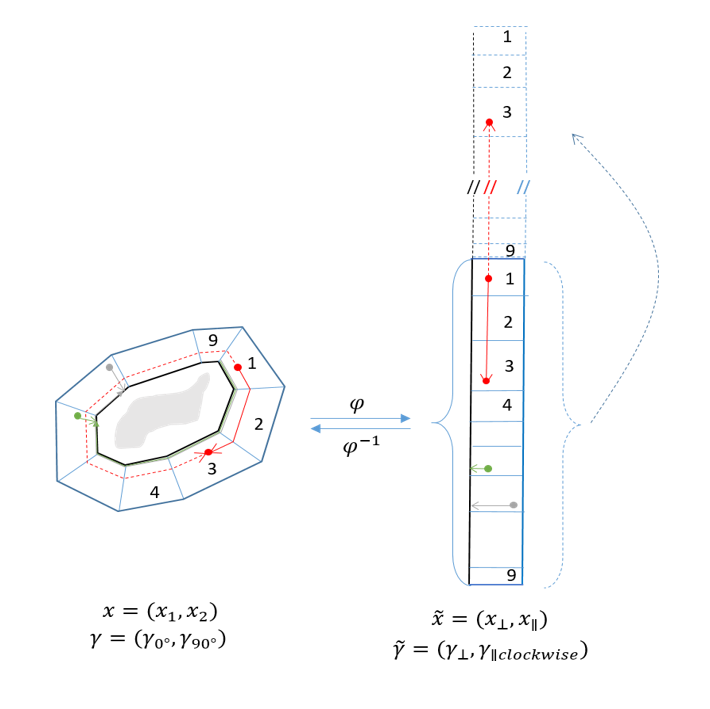


Supplementary Figure S5. Schematic representation of the algorithm used to unfold the domain area problem prior to variography and kriging. ϕ is the projection function; $x,\tilde{x}$ denote respectively the data coordinates in the geographical space and in the projected space; γ,$\tilde{\gamma}$ denote respectively variogram in the geographical space and in the projected data.


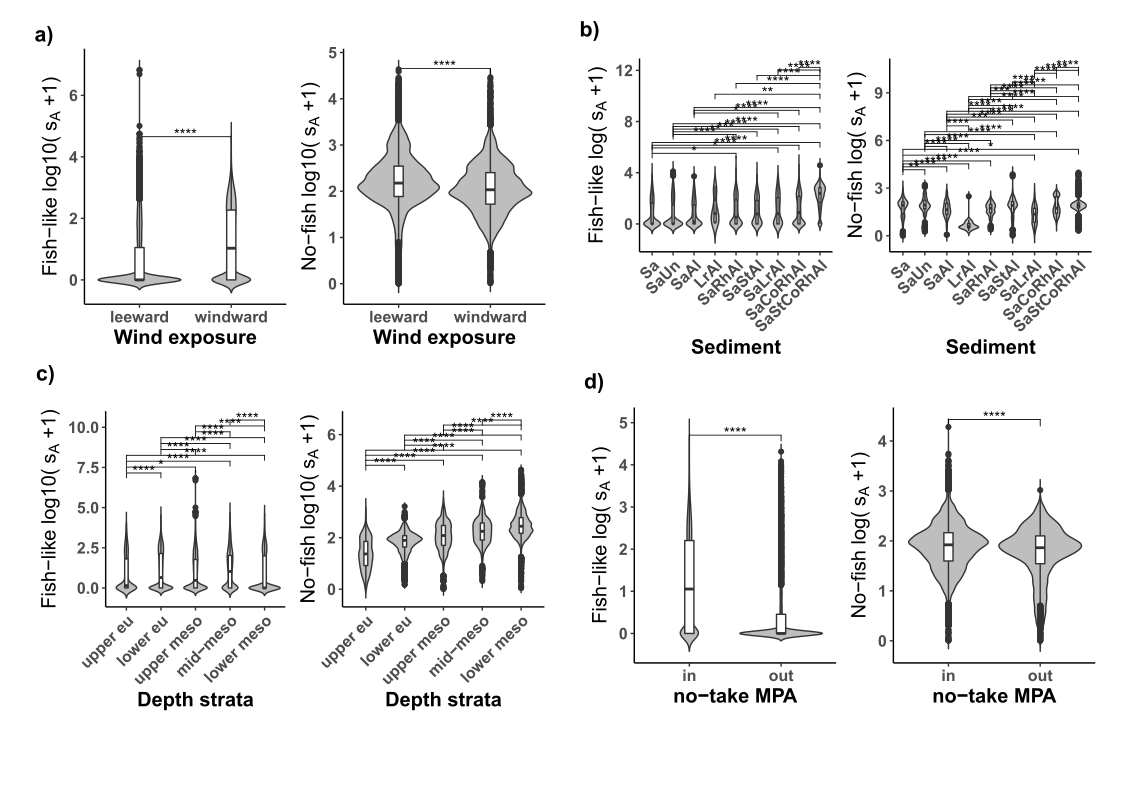


Supplementary Figure S6. Violin plot containing boxplot representing the median (horizontal bar), interquartile range, whiskers and outlying points of the fish-like and no-fish biomass (log10(sA+1)) according to (a) the position around FNA, windward vs. leeward; (b) the sediment type; (c) the bottom depth range: upper euphotic (0-20 m), lower euphotic (20-40 m), upper mesophotic (40-60 m), mid-mesophotic (60-80 m), lower mesophotic (80-100 m); and (d) the protection level, i.e., in vs out the no-take zone. The stars indicate the level of significance with (****), (***), (**), and (*), corresponding to p value < 0.0001, < 0.001, < 0.01 and < 0.05, respectively.


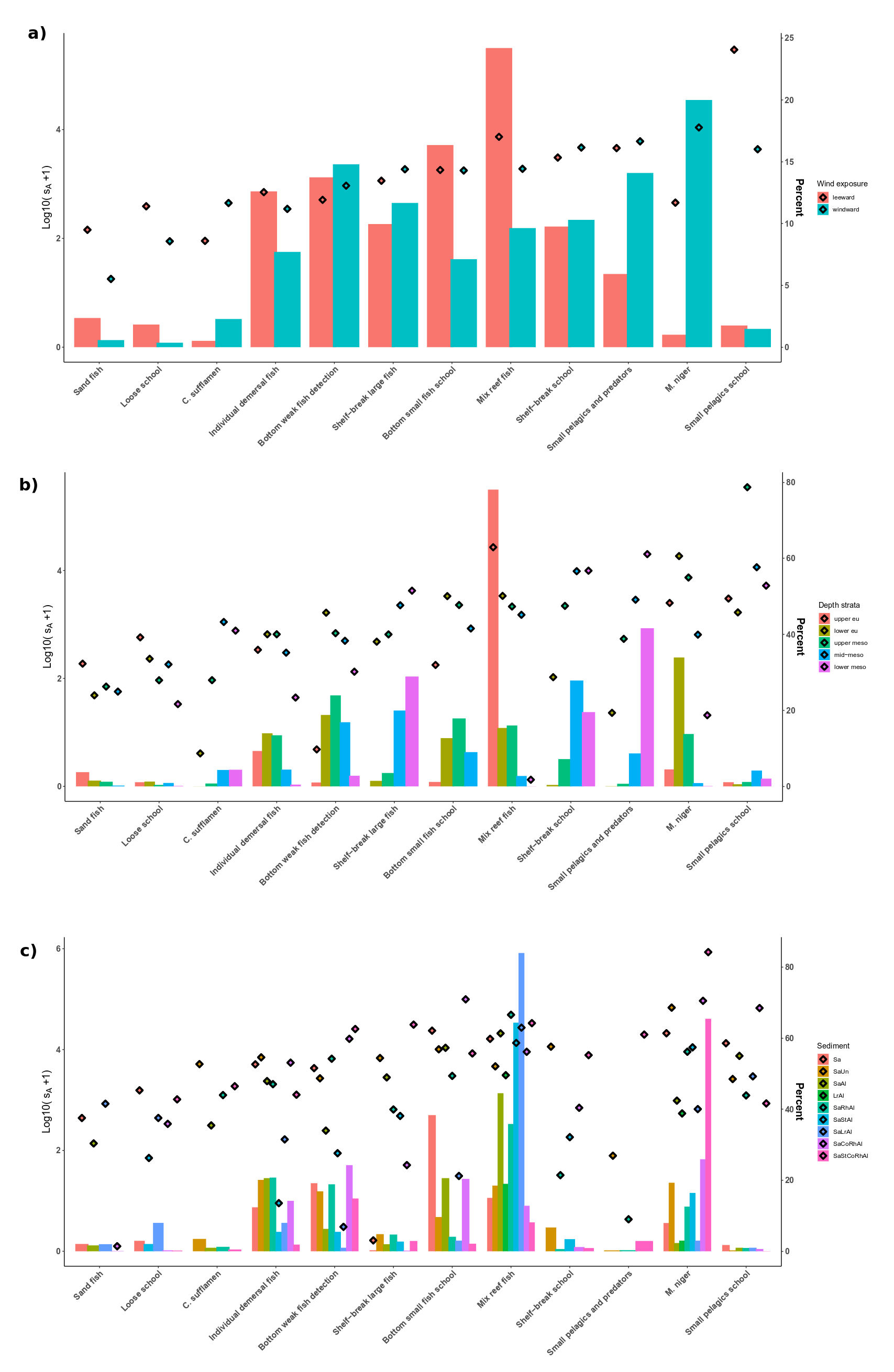


Supplementary Figure S7. Barplot representing the percentage of ESDU with presence of each assemblage according to (a) the wind exposure (leeward vs. windward); (b) the depth strata, and (c) the sediment. The mean acoustic biomass of each group (log10(s_A_+1)) per ESDU (i.e. the total acoustic biomass normalised by the number of ESDU in each side) is represented by diamonds (right y-axis).
